# Trail Resistance Induces Epithelial-Mesenchymal Transition and Enhances Invasiveness by Suppressing PTEN via miR-221 in Breast Cancer

**DOI:** 10.1371/journal.pone.0099067

**Published:** 2014-06-06

**Authors:** Haiji Wang, Chunyuan Xu, Xiaoli Kong, Xiaoyan Li, Xiangnan Kong, Yu Wang, Xia Ding, Qifeng Yang

**Affiliations:** 1 Department of Oncology, Qilu Hospital, Shandong University, Jinan, Shandong Province, China; 2 Department of Oncology, Affiliated Hospital of Qingdao University Medical College, Qingdao, Shandong Province, China; 3 Department of Breast Surgery, Qilu Hospital, Shandong University, Jinan, Shandong Province, China; 4 Department of Oncology, Qingdao Municipal Hospital, Qingdao, Shandong Province, China; 5 Department of Obstetrics and Gynecology, Qilu Hospital, Shandong University, Jinan, Shandong Province, China; Rajiv Gandhi Centre for Biotechnology, India

## Abstract

Tumor necrosis factor-related apoptosis-inducing ligand (TRAIL) can selectively induce apoptosis of cancer cells and is verified effective to various cancers. However, a variety of breast cancer cell lines are resistant to TRAIL and the mechanisms of resistance are largely unknown. In our present experiment, we successfully utilized breast cancer cell line MDA-MB-231 to establish TRAIL-resistant cell line. We found resistance to TRAIL could induce epithelial-mesenchymal transition (EMT) and enhance invasiveness. We further demonstrated PTEN was down-regulated in TRAIL-resistant cells. Silencing miR-221, PTEN expression was up-regulated, the process of EMT could be reversed, and the ability of migration and invasion were correspondingly weakened. We also demonstrated knockdown of miR-221 could reverse resistance to TRAIL partially by targeting PTEN. Our findings suggest that resistance to TRAIL could induce EMT and enhance invasiveness by suppressing PTEN via miR-221. Re-expression of miR-221 or targeting PTEN might serve as potential therapeutic approaches for the treatment of Trail-resistant breast cancer.

## Introduction

Breast cancer has become the first common malignancy in women in developed and developing countries. About 1.3 million women are diagnosed with breast cancer each year over the world [1]. Surgery is the important therapeutic method of breast cancer. Chemotherapy, radiotherapy and endocrine therapy also play important roles in breast cancer. Although with comprehensive therapy, there are about 0.5 million women patients died of breast cancer each year due to recurrence, metastasis and resistance to therapy [2]. Therefore, more effective therapeutic strategies are required to improve treatment outcomes for breast cancer patients.

Tumor necrosis factor-related apoptosis-inducing ligand (TRAIL), a member of the TNF super family, can selectively induce apoptosis of cancer cells and has emerged as a promising anticancer agent [3]. While using alone or combination with other agents, it is shown encouraging outcomes with no apparent toxicity in pre- and clinical trials and it can overcome multi-cytotoxic and endocrine drugs resistance [4]–[6]. But recent studies indicate that most varieties of cancer cells, including ovarian cancer, urothelial cancer, pancreatic cancer, colon cancer, prostate cancer [7]–[10] and half of breast cancer cell lines (all of the ER positive breast cancer cell lines, most Her-2 positive cell lines and few triple negative cell lines that exhibit epithelial phenotype), are resistant to the apoptotic effects of TRAIL [11]. Moreover, it is indicated that resistance to TRAIL can enhance the invasiveness of variety of cancer cells [12]. The clinical utility of TRAIL is hence limited by this development of resistance in the treatment of human malignancies, it is very important to reveal the mechanisms of resistance to TRAIL.

The mechanisms of resistance to TRAIL and the enhancement of invasiveness are not fully understood. It has been reported that resistance to TRAIL occurs due to several factors, including low expression levels of TRAIL-R1 (or death receptor (DR) 4) and TRAIL-R2(or DR5), elevated levels of TRAIL-R3 (or decoy receptor (DcR) 1) and TRAIL-R4 (or DcR2), increased levels of negative regulators of apoptosis 9 such as cFLIP, etc. [13]–[15]. Moreover, the up-regulation of cell survival and proliferation pathways, through mitogen-activated protein kinases (MAPK) and nuclear factor-κB (NF-κB) activations, are crucial for the resistance [16], [17]. In this study, we reported that TRAIL-resistant breast cancer cells had the enhanced invasiveness and undergone epithelial to mesenchymal transition (EMT) by down-regulation of PTEN via miR-221.

## Materials and Methods

### Reagents and antibodies

Rabbit anti-N-cadherin and anti-fibronectin was from Abcam (Cambrigeshire, England). 24-well plates, transwell chambers and matrigel were purchased from BD Biosciences, Bedford, MA, USA. Rabbit anti-vimentin, anti-Snail and anti-PTEN were from Cell Signaling (Beverly, MA, USA). Mouse anti-β-actin was supplied by Sigma (St. Louis, MO, USA). Anti-mouse IgG horseradish peroxidase (HRP) antibody was from ZhongShan Goldenbridge (Beijing, China). Pro-lighting HRP agent for western blotting detection was from Tiangen Biotech Co. Ltd (Beijing, China). Cell lysis buffer for western blotting was purchased from Beyotime Institute of Biotechnology (Jiangsu, China). Other reagents were from Sigma–Aldrich (St. Louis, MO, USA) unless specifically described.

### Cells and culture conditions

The breast cancer cell line MDA-MB-231 and mouse fibroblast cell line NIH3T3 were obtained from American Type Culture Collection, ATCC, Rockville, MD, USA. They are routinely cultured in Dulbecco's Modified Eagle's Medium (DMEM, obtained from Gibco-BRL, Rockville, IN, USA), containing 10% fetal bovine serum (FBS, supplied by Haoyang biological manufacture, Tianjin, China), 100 U/ml penicillin and 100 U/ml streptomycin at 37°C with 5%CO_2_ in a humidified incubator.

### Production of recombinant human TRAIL (rhTRAIL)

RhTRAIL was produced according to the method established previously [18], [19]. Briefly, we use a M15 pRep4 bacteria with pQE9 expression plasmid (Qiagen), which could code for the sequence MRGSHHHHHHGSEQKLISEEDLNLQ (His_6_-Myc) followed by amino acids 95-281 of human TRAIL [20]. When growing to the density of 0.7 at 600 nm, bacteria were induced with 50 µg/ml isopropyl-1-thio-β-D-galactopyranoside (IPTG). After 18 h at 37°C, the bacteria was suspended with B-PER lysis buffer (Thermo Fisher Scientific, Rockford, IL, USA) according to the manufacturer's guidance. The filtered supernatant was washed after centrifugation with 2 column volumes of TBS (10 Mm Tris-HCL pH 7.4, 140 mM NaCl) containing 10 mM imidazole followed by 2 column volumes of TBS with 50 mM imidazole. After that, the production was eluted with TBS, containing 0.5 mM imidazole, and dialyzed in a dialysis bag. After confirmed by SDS-PAGE, rhTRAIL was stored at -80°C and was used within 8 months to assure its effectiveness.

### Establishment of TRAIL-resistant cell line

Breast cancer cell line MDA-MB-231 was cultured in DMEM with 10%FBS. Initiative dosage of 2 ng/ml rhTRAIL was added into the culture medium. The cells were routinely cultured. After 2∼3 weeks, assuring that the cells were passaged more than 3 times, the dosage of rhTRAIL was gradually increased and was kept culturing as previously described. Ultimately, the final dosage of rhTRAIL in the culture medium was 4 µg/ml, which was more than 10 times of median lethal dose. This final concentration was maintained over 2 months to keep the resistance of cells. rhTRAIL was removed from the medium at least one week before the TRAIL-resistant cells were used.

### Cell viability assay

Cell viability assay was performed with the 3-(4, 5-dimethylthiazol-2-yl)-2, 5-diphenyltetrazolium bromide (MTT) assay. Cells were planted at density of 2,000 cells per well in 96-well plates. After incubating in the incubator overnight, rhTRAIL at gradient concentration (from 1 ng/ml to 1,000 ng/ml) was added into the medium. After another 24∼48 hours incubation at 37°C, 20 µl of MTT (5 mg/ml in PBS) was added into each well. Then cells were incubated for another 4 hours. The suspensions were carefully removed and then 100 µl of dimethyl sulfoxide (DMSO) was added into each well. The plates were gently shaken for 10 minutes and the absorbancy was measured at 490 nm with Microplate Reader (Bio-Rad, Hercules, CA, USA).

### Invasion and migration assay

To perform invasion and migration assay, we use the technique of transwell. Conditional medium was obtained from supernatant of NIH3T3. For invasion assay, the invasion chambers in 24-well plate coated with 50 µl matrigel diluted in DMEM (serum free) were incubated at 37°C for 2 hours at first. Being the chemotactic factor, 700 µl of conditional medium were added to each lower compartment. While the upper compartments of the chambers with or without matrigel (no matrigel for migration assay) were covered with 200 µl DMEM without serum containing 1×10^5^ cells. Put the plate into the incubator. After 12 hours for migration assay and 20 hours for invasion assay, the chambers were washed with PBS for 3 times, fixed with methanol and stained with hematoxylin-eosin. The noninvasive cells were erased with cotton swabs. After drying out, the cells that migrated through the membrane and stuck to the lower surface of the membrane were counted under a light microscope.

### SiRNA and transfection

To knockdown the expression of PTEN in MDA-MB-231 cells, we transfected small interfering RNA (siRNA) of PTEN and NC (obtained from Genepharma, Shanghai, China) with lipofectamine 2000 (Invitrogen) into MDA-MB-231 cells according to the manufacturer's protocol. Similarly, the inhibitor of miR-221, miR-21 and the corresponding negative control (NC) (supplied by Genepharma, Shanghai, China) were transfected into TRAIL-resistance cells to knowdown the expression of miR-221 and miR-21. Transiently transfected cells were harvested after 24 h for mRNA and 48∼72 h for MTT, Transwell and protein analysis.

### RNA extraction and quantitative real-time RT-PCR analysis (qRT-PCR)

Total RNA was extracted from cells with TRIzol reagents (Invitrogen) according to the manufacturer's protocol. Measure the absorbance at 260 nm to determine the concentration of total RNA. 1 µg of RNA of each sample was reversely transcribed to cDNA by One Step Primerscript miRNA cDNA Synthesis kit (Takara, Dalian, China) and PrimeScript RT reagent Kit (Takara, Dalian, China). QRT-PCR was carried out by the SYBR green Premix Ex Taq II (Takara) with Applied Biosystems StepOne Plus Real-Time PCR System (Applied Biosystems, Carlsbad, CA, USA). The expression of U6 was used as endogenous control for analysis of miRNAs expression and GAPDH was used as endogenous control for analysis of other mRNA expression. The primers were synthesized by Sangon Biotech, Shanghai, China. Relative quantification was analyzed with the comparative △CT values.

### Western blot analysis

Cells were washed for 3 times with cold PBS before being lysed with radio immunoprecipitation assay (RIPA) buffer (PBS, 1% NP40, 0.1% SDS, 5 mM EDTA, 0.5% sodium deoxycholate, 1 mM sodium orthovanadate) with protease inhibitors on ice. Measure the concentration of each protein sample with Microplate Reader. Identical amounts of each protein (50 µg) were separated by SDS-PAGE gels and electro-transferred to PVDF membranes (ImmobilonP, Millipore, Bedford, MA, USA). Block the PVDF membranes in 5% non-fat milk resolved in Tris-buffered saline for one hour before incubating them at 4°C overnight with their primary antibody. Then the membranes were blocked with their corresponding horseradish peroxydase conjugate secondary antibody. Signals were detected with enhanced chemiluminescence. β-actin was used as the control. All the experiments were repeated at least in triplicate to confirm the findings.

### Statistical analysis

All experiments were repeated at least 3 times independently. Statistical analysis was performed by SPSS V18.0. Significance was determined by paired T test. The results were shown as mean ± standard deviation (SD). P<0.05 was defined as significant difference, while P<0.01 as dramatically significant difference.

## Results

### TRAIL-resistant MDA-MB-231 cells appeared morphological transformation

**Figure 1:**
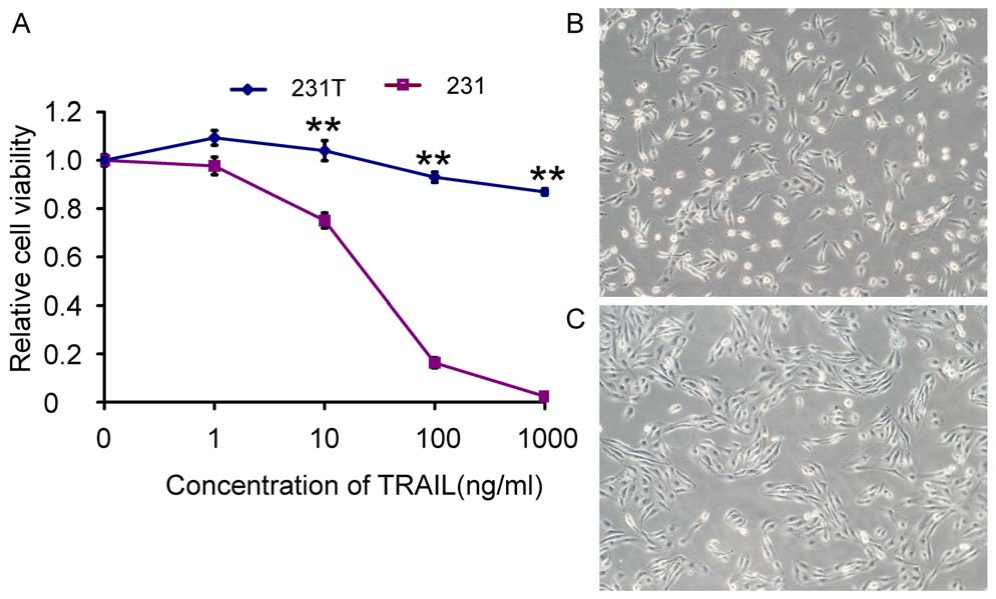
TRAIL-resistant cells (231T) were resistant to TRAIL and had different morphology from MDA-MB-231 cells (231). **A**. Sensitization of 231 cells and 231T cells to TRAIL at gradient concentrations was measured by MTT assay. Points represented the average of three independent experiments. Bars stood for SD; *P<0.05; **P<0.01; B and C. Resistance to TRAIL induced morphological change. **B**: 231 cells. **C**: 231T cells. Cells were observed under a light microscope.

We successfully established the TRAIL-resistant MDA-MB-231 cells (231T) as described above. They were obviously resistant to rhTRAIL comparing with parental MDA-MB-231 (231) by MTT assay (**Figure 1A**). Furthermore, the morphology of 231T cells was different with 231 cells under a light microscope. They became longer and richer than 231 cells, and emerged a cluster growth, just like the ear of wheat (**Figure 1B and C**).

### Resistance to TRAIL led to EMT

**Figure 2:**
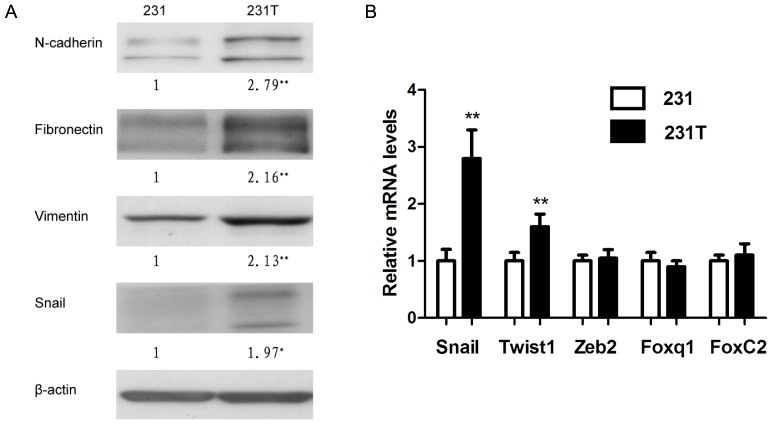
Resistance to TRAIL induced EMT. **A**.The mesenchymal markers, including N-cad, fibronectin, vimentin, and Snail were examined by western blot analysis. β-actin was used to confirm equal loading of samples. **B**. The mRNA expressions of transcriptional factors including Snail, Twist1, Zeb2, Foxq1 and FoxC2. *P<0.05; **P<0.01.

Once EMT is familiar with chemotherapeutics-resistance cancer cells, combining with the morphological transformation and documents, we hypothesized that in 231T cells, EMT also existed. So we then examined the EMT markers in 231 cells and 231T cells by western blot. Because in MDA-MB-231 cells, the expression of E-cadherin and other epithelial markers are very low, almost undetectable, we examined some mesenchymal markers whose elevation also could indicate the occurrence of EMT. The results showed that the mesenchymal markers, including N-cadherin, fibronectin and vimentin, were all over-expressed in 231T cells. Moreover, the expression of Snail, a transcriptional repressor of E-cadherin, was also arose (**Figure 2A**). The mRNA levels of some transcription factors were also examined by real-time PCR (**Figure 2B**). The results showed that Snail and Twist were up-regulated in 231T cells, and other factors were almost no change. These results verified that resistance to TRAIL could induce EMT.

### Resistance to TRAIL increased migration and invasion

**Figure 3:**
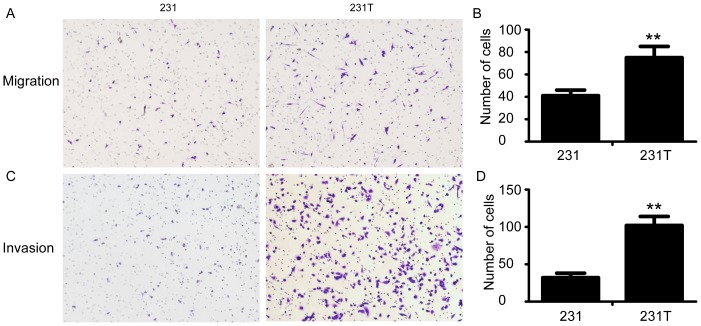
Resistance to TRAIL enhanced cell invasiveness. **A**. Migration assay of 231 cells and 231T cells. **C**. Invasion assay of 231 and 231T cells. Cells that passed through the membrane were counted in 10 representative fields. Diagrams for migration (**B**) and invasion (**D**) were shown, respectively. Data was shown as mean ± SD. *P<0.05; **P<0.01.

The acquisition of EMT can enhance the abilities of migration and invasion of cancer cells. Hence the western blot results had shown that resistance to TRAIL could induce EMT, we then evaluated both migration and invasion abilities by Transwell assay. After counting under light microscope and statistical calculation, we found that 231T cells had enhanced capabilities of both migration and invasion comparing with 231 cells (p<0.01) (**Figure 3**).

### Down-regulation of PTEN induced TRAIL resistance, EMT and enhanced migration and invasion

**Figure 4:**
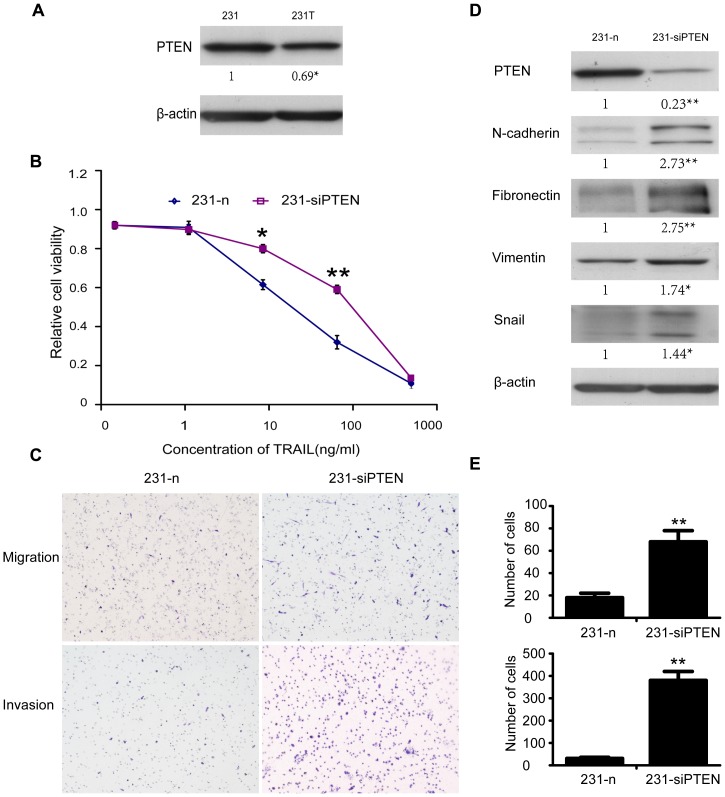
Down-regulation of PTEN resulted in resistance to TRAIL of 231 cells, EMT and enhancement of invasiveness. **A**. PTEN was down-regulated in 231T cells compared with 231. **B**. MDA-MB-231 cells transfected with siPTEN and its corresponding NC were named as 231-siPTEN and 231-n respectively. Tolerance of 231-n cells and 231-siPTEN cells to different concentrations of TRAIL was examined by MTT assay. Points represented the average of three independent experiments. Bars stood for SD; **C**. Migration and invasion assay of 231-n cells and 231-siPTEN cells. Count the cells in 10 representative fields under a light microscope. **D**. N-cadherin, fibronectin, vimentin and Snail of 231-n and 231-siPTEN cells were detected by western blot assay. Also β-actin was used as control. **E**. Summary graphs for migration and invasion, respectively. Data were shown as mean ± SD. *P<0.05; **P<0.01.

To clarify the molecular mechanisms of resistance to TRAIL and the induction of EMT, we detected PTEN in 231T cells and found it was down-regulated in 231T cells (**Figure 4A**). We further interfered the expression of PTEN in 231 cells. Then MTT assay, western blot and Transwell test were all performed with cells transfected with siRNA of PTEN (231-siPTEN) and its negative control (231-n). Knockdown of PTEN resulted in insensitivity to TRAIL of 231 cells (**Figure 4B**). Results of western blot exhibited that the EMT markers and transcriptional marker Snail tested in the previous experiments were all up-regulated (**Figure 4D**). Moreover, cells abilities of migration and invasion were enhanced once again after interference the expression of PTEN (**Figure 4C and E**). So we concluded that PTEN down-regulation could lead to TRAIL-resistance, EMT, enhancement of migration and invasion.

### PTEN was regulated by miR-221 in TRAIL-resistant MDA-MB-231 cells

**Figure 5:**
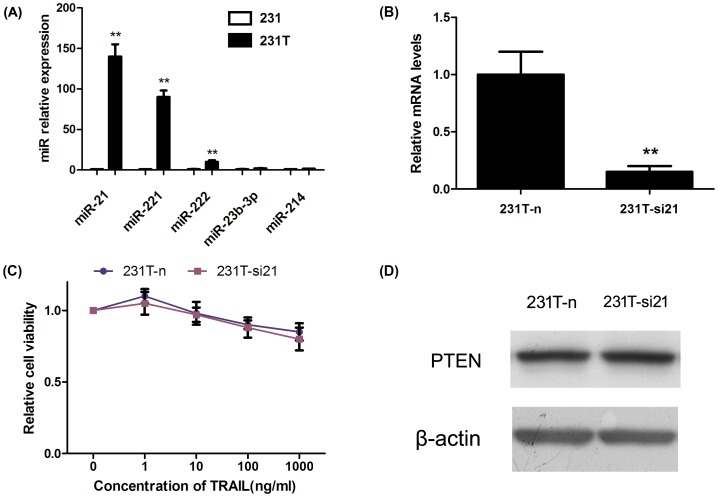
MiR-21 was over-expressed in 231T cells but not the key regulator in TRAIL-resistance. **A**. Several miRNAs were detected in 231 and 231T cells. miR-21 and miR-221 were statistically up-regulated in 231T cells. **B**. miR-21 was silenced by inhibitor in 231T cells. **C**. Silence of miR-21 didn't reverse the resistance of 231T cells measured by MTT assay. **D**. After silencing miR-21, expression of PTEN didn't change. *P<0.05; **P<0.01.

In order to explore the regulator of PTEN, we detected miR-21, miR-221, miR-222, miR-23b-3p and miR-214 expression based on data mining and miRNA prediction. The expression of miR-23b-3p and miR-214 was not different between 231 cells and 231T cells. We found that miR-21 was over-expressed obviously in 231T cells, about 140 times compared with 231 cells, which is the highest of the miRNAs we detected (**Figure 5A**). Then we inhibited miR-21 in 231T cells (**Figure 5B**), but the cells were not turn to be sensitive to TRAIL (**Figure 5C**). Furthermore, the expression of PTEN was equal to 231T cells (**Figure 5D**). Therefore, we infer that miR-21 was not the key regulator of PTEN and it was not related with TRAIL-resistance.

**Figure 6:**
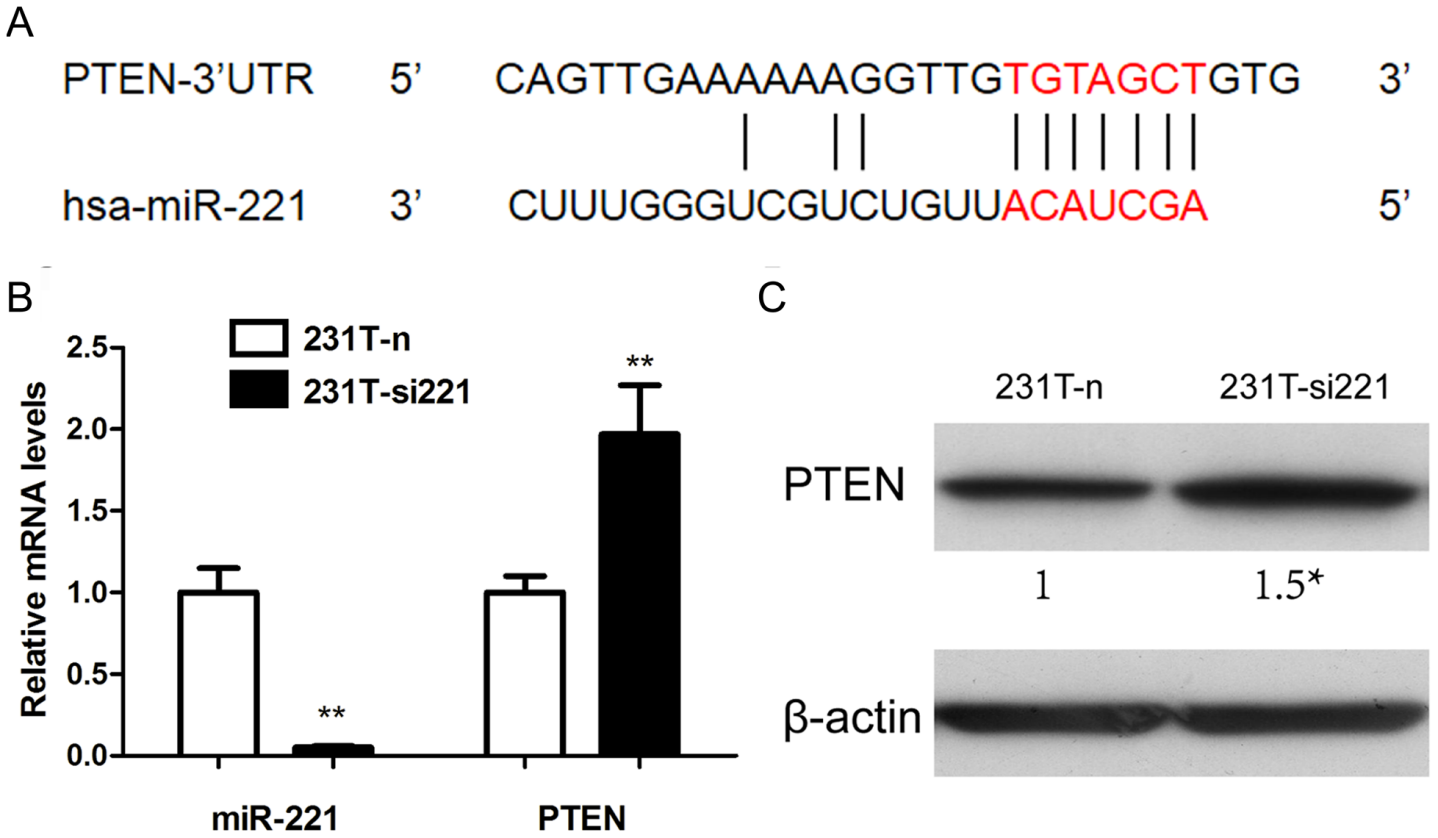
PTEN was the target gene of miR-221. **A**. miR-221 was predicted to regulate PTEN. **B**. After silencing miR-221, the mRNA level of PTEN was significantly up-regulated detected by real-time PCR. **C**. The corresponding change of PTEN in 231T-n and 231T-si221 cells was detected by western blot assay. *P<0.05; **P<0.01.

We further analyzed miR-221, another PTEN possible regulator (**Figure 6A**). In 231T cells, miR-221 was over-expressed, which was over 90 times in contrast with 231 cells, next only to miR-21(**Figure 5A**). Since miR-21 might not be the key regulator of TRAIL-resistance, we performed knockdown of miR-221 in 231T cells by transfection (231T-si221), and confirmed the efficiency of transfection by real-time RT-PCR contrasting with cells transfected with negative control (231T-n). After transfection, the mRNA level and protein level of PTEN were both significantly up-regulated (p<0.05) (**Figure 6B and C**). These data indicated that resistance to TRAIL could induce the up-regulation of miR-221 and PTEN might be the target gene of miR-221.

### Inhibition of miR-221 reversed resistance to TRAIL, EMT and weaked migration and invasion

**Figure 7:**
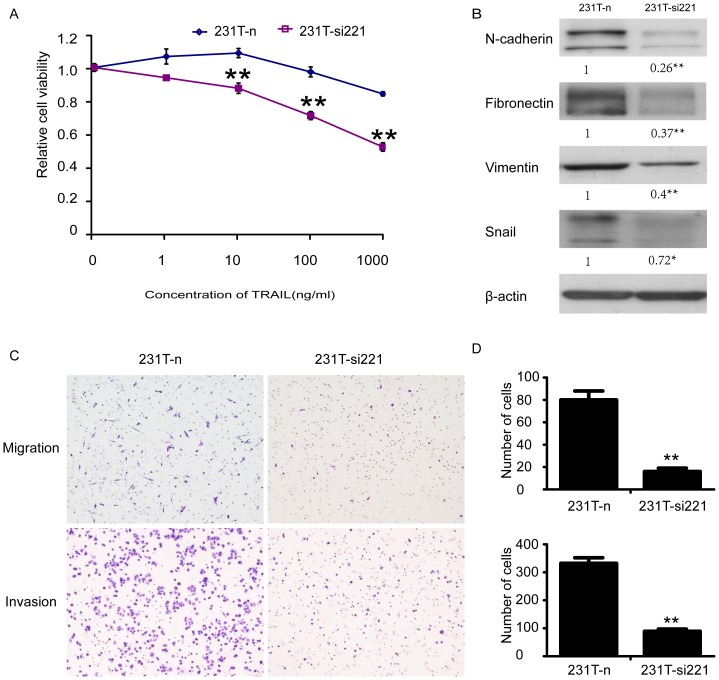
MiR-221 knockdown could sensitize 231T cells to TRAIL, reverse EMT and reduce cell mobility. **A**. Cultured 231T-n and 231T-si221 cells with different concentrations of TRAIL in 96-well plates. 48 hours later examined their sensitivity to TRAIL by MTT assay. Points represented the average of three independent experiments. Bars stood for SD; **B**. The mesenchymal markers, N-cadherin, fibronectin, vimentin and Snail expressed in 231T-n and 231T-si221 cells were detected by western blot assay. β-actin was used as control; **C**. Migration and invasion assay of 231T-n cells and 231T-si221 cells. Staining the migrated cells with hematoxylin–eosin and count them in 10 representative fields under a light microscope. **D**. Summary graphs for migration and invasion were also shown, respectively. Data was presented as mean ± SD. *P<0.05; **P<0.01.

To clarify the role of miR-221 in resistance to TRAIL, we performed drug sensitivity test of 231T-si221 cells and 231T-n cells using MTT assay. Data showed that knockdown of miR-221 could recover sectional sensitivity to TRAIL (**Figure 7A**). Then we detected those EMT markers (including N-cadherin, fibronectin, vimentin) again (**Figure 7B**). And found that those mesenchymal markers and transcriptional marker Snail were down-regulated, showing that EMT was reversed. Moreover, we performed Transwell test of 231T-n and 231T-si221 cells, and discovered that after inhibition of miR-221, the abilities of migration and invasion were both weakened (**Figure 7C and D**). All of these results accounted for that miR-221 participated in the resistance to TRAIL, EMT, migration and invasion of MDA-MB-231 cells.

## Discussion

Chemotherapy plays very important role in treatment of breast cancer, however, many patients are resistant to chemotherapeutic agents and chemotherapy has few effects on these patients [21]. To overcome this situation, new drugs are urgent to be developed and utilized in treatment of breast cancer. TRAIL is considered to be a promising anticancer agent, which can selectively induce apoptosis of cancer cells. It is reported to be effective with almost no side effect for triple negative breast cancer and has been testified in pre- and clinical trials, especially for those basal-like cell lines with mesenchymal phenotype [22]. But recent studies indicated that half of the breast cancer cells were resistant to TRAIL [11]. Understanding the mechanisms of resistance to TRAIL can provide meaningful guidance for clinical treatment of breast cancer and give more effective therapeutic methods for those patients who are resistant to TRAIL.

In our study, we successful established TRAIL-resistant MDA-MB-231 cells (231T) and we found that resistance to TRAIL could lead to EMT. The processes of EMT include down-expression of epithelial markers (such as E-cadherin), and up-regulation the expression of mesenchymal markers (such as N-cadherin, fibronectin, vimentin) [23], [24]. In 231T cells, mesenchymal markers, including N-cadherin, fibronectin and vimentin, were all over-expressed compared with parental MDA-MB-231 (231) cells. The expression of Snail was also arised. It has been reported that several drug-resistance cancer cells can enhance invasiveness by inducing EMT [25], [26]. In our present study, resistance to TRAIL also increased abilities of migration and invasion measured by Transwell assay (p<0.01). After counting under light microscope and statistical calculation, we found that 231T cells had enhanced capabilities of both migration and invasion comparing with 231 cells (p<0.01). Based on our present findings, we can conclude that Trail resistance induces epithelial-mesenchymal transition and enhances invasiveness in breast cancer cells.

As a tumor suppressor gene, PTEN has been well documented that it played a vital role in the inhibition of progress via regulating various cell survival pathways, including PI3K/AKT and mitogen-activated kinase (MAPK) pathway [27], [28]. And down-regulation of PTEN is important for the development of multi-drugs resistance, including that to TRAIL [29]–[31]. In addition, it was reported that PTEN was correlated with EMT, which could enhance the abilities of invasion and migration [32], [33]. Consistently, in our present study, we detected PTEN in 231T cells and found it was down-regulated, and once silencing the expression of PTEN, 231 cells were resistant to TRAIL and the abilities of migration and invasion were both enhanced.

As reported in the literature, PTEN was regulated by several MicroRNAs (miRNAs), such as miR-21, miR-221, miR-23b-3p, miR-214, and so on [34]-[37]. MicroRNAs (miRNAs), which are small, non-coding RNA molecules, are reported to regulate crucial biological processes by targeting their specific mRNAs[38]. MiRNAs play an important role in several physiological processes in cancer cells, including development, proliferation, migration and invasion, differentiation and apoptosis, via regulating their targeting genes [39], [40]. In our present study, we detected the expression of several miRNAs based on data mining and miRNA prediction. Of them, miR-21 was up-regulated in 231T cells, but while inhibit its expression, the cells were still resistant to TRAIL and the expression of PTEN was also not changed. So we concluded that miR-21 was not the key regulator of PTEN in 231T cells. Then the effect of miR-221, which was next only to miR-21 in 231T cells, was examined. By using PicTar, miRanda and TargetScan database, miR-221 was predicted to regulate PTEN. After silencing miR-221, PTEN was obviously up-regulated on both mRNA and protein level, the same as papers reported before [35], [41]. Thus we expected that miR-221 directly regulated PTEN. While it was confirmed by our experiment that miR-221 acted as an oncomiRNA to induce EMT and boost malignancy of TRAIL-resistant cancer cells, accordance with most documents reported before [35], [42], [43]. Silencing miR-221, the cells were sensitive to TRAIL again. Afterwards, the process of EMT reversed, and the abilities of migration and invasion were correspondingly weakened. So we verified that in TRAIL-resistant cells, miR-221 improved migration and invasion and induced EMT by targeting PTEN. Our results clearly indicated that miR-221 contributed to TRAIL-resistance by targeting PTEN, in agreement with reports in other cancers [43], [44]. However, knockdown of miR-221 could not reverse resistance completely, so as to PTEN. Therefore we think that during the process of TRAIL-resistance, there are other factors play a part, which is waiting for more researches.

Our study indicated that TRAIL resistance induced EMT. However it was also probable that TRAIL induced cell death, except for few cells which exhibited a different cellular equipment leading to an EMT phenotype. These cells were selected and the population acquired this new phenotype. Moreover, this present study choose only one breast cancer cell line to investigate the mechanisms of TRAIL-resistance. Afterwards, researchers will establish more TRAIL-resistant cells using other kinds of breast cancer cell lines to further verify our findings.

In conclusion, we established TRAIL-resistant MDA-MB-231 cell line and found resistance to TRAIL could induce EMT and enhance invasiveness. MiR-221 could modulate sensitivity of cancer cells to TRAIL through PTEN. Our findings suggest that re-expression of miR-221 or targeting PTEN might serve as potential therapeutic approaches for the treatment of Trail-resistant breast cancer.
