# Correction: Trail Resistance Induces Epithelial-Mesenchymal Transition and Enhances Invasiveness by Suppressing PTEN via miR-221 in Breast Cancer

**DOI:** 10.1371/journal.pone.0214433

**Published:** 2019-03-21

**Authors:** Haiji Wang, Chunyuan Xu, Xiaoli Kong, Xiaoyan Li, Xiangnan Kong, Yu Wang, Xia Ding, Qifeng Yang

After publication of this article [1], concerns were raised about the B-actin panel of Fig 2A being similar to B-actin panel of Fig 7B and the 231T panel in Fig 3A containing a region of overlap with 231-T panel in Fig 7C. The authors made an error when processing the images. In addition, the primary data underlying results in this article were not included with the published article.

With this Correction, the authors provide updated Figs 4, 6 and 7, along with all original raw data (in Supporting Information S1 File).

A member of PLOS ONE’s Editorial Board confirmed that the new Figures and provided data support the results and conclusions of the published article.

The authors apologize for the errors in the published article.

**Fig 4:**
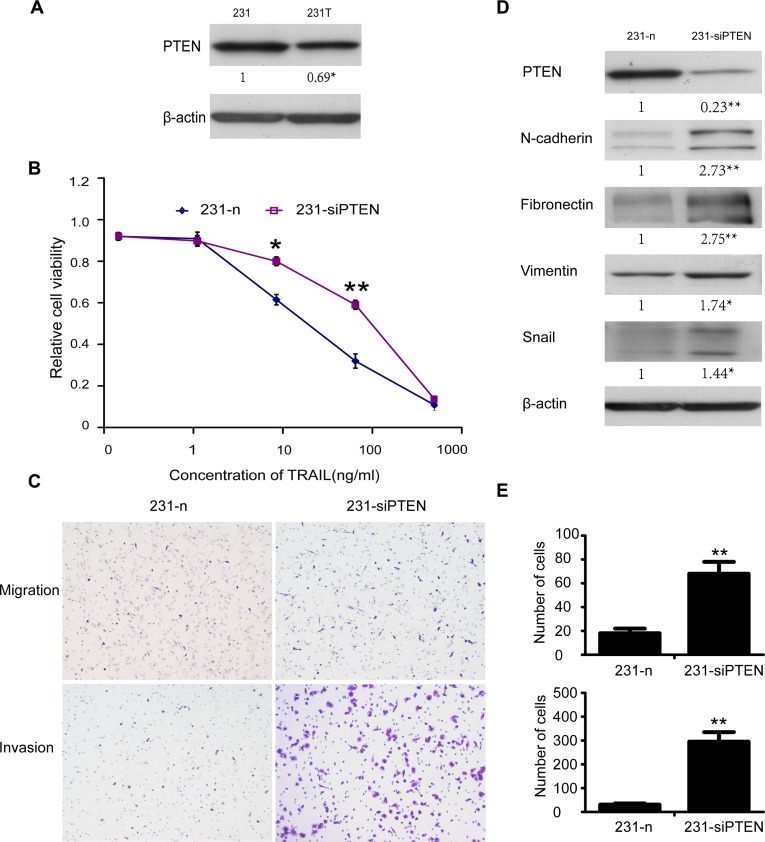
Down-regulation of PTEN resulted in resistance to TRAIL of 231 cells, EMT and enhancement of invasiveness. **A.** PTEN was down-regulated in 231T cells compared with 231. **B.** MDA-MB-231 cells transfected with siPTEN and its corresponding NC were named as 231-siPTEN and 231-n respectively. Tolerance of 231-n cells and 231-siPTEN cells to different concentrations of TRAIL was examined by MTT assay. Points represented the average of three independent experiments. Bars stood for SD; **C.** Migration and invasion assay of 231-n cells and 231-siPTEN cells. Count the cells in 10 representative fields under a light microscope. **D.** N-cadherin, fibronectin, vimentin and Snail of 231-n and 231-siPTEN cells were detected by western blot assay. Also b-actin was used as control. **E.** Summary graphs for migration and invasion, respectively. Data were shown as mean 6 SD. *P,0.05; **P,0.01.

**Fig 6:**
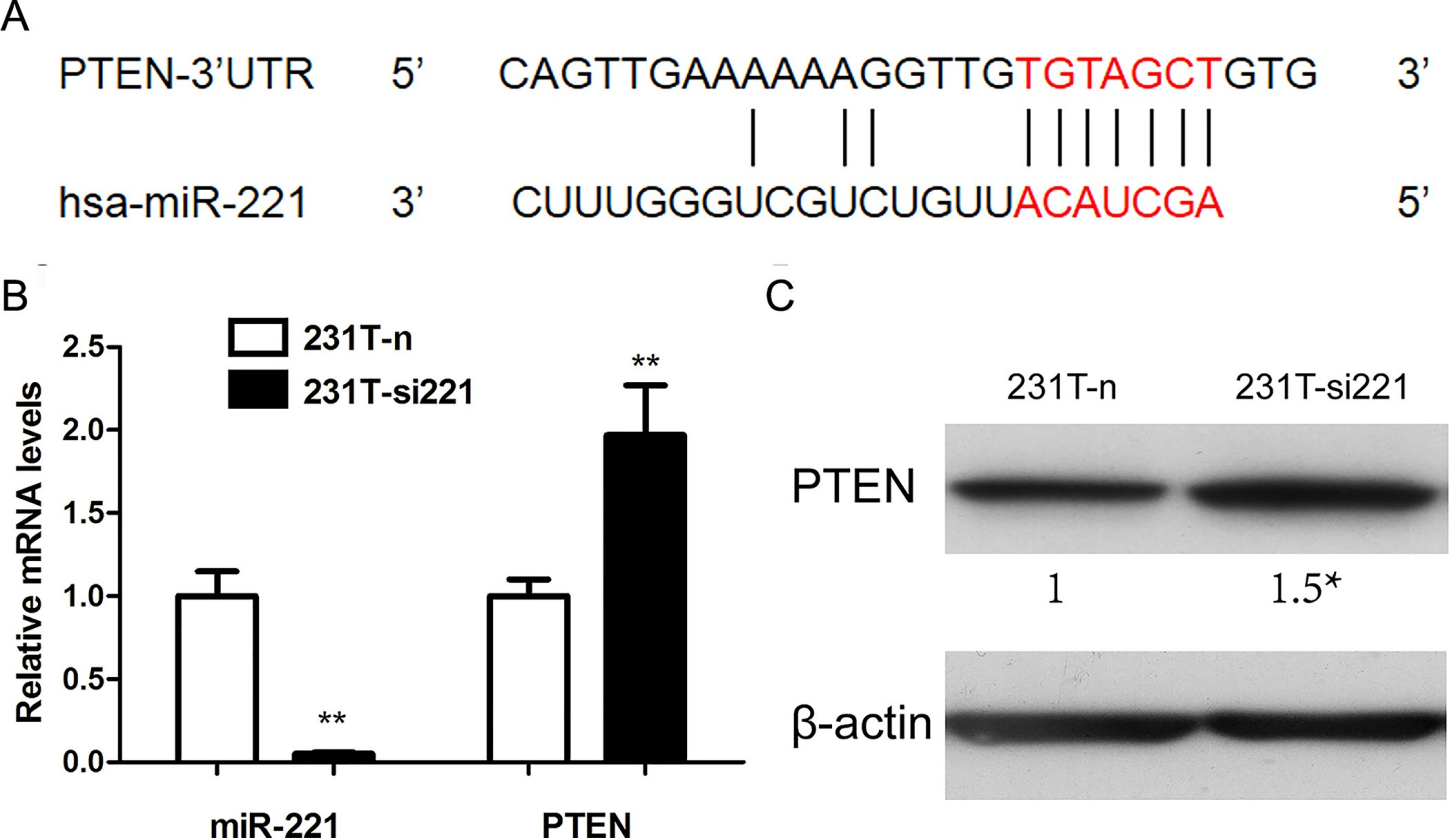
PTEN was the target gene of miR-221. **A.** miR-221 was predicted to regulate PTEN. **B.** After silencing miR-221, the mRNA level of PTEN was significantly up-regulated detected by real-time PCR. **C.** The corresponding change of PTEN in 231T-n and 231T-si221 cells was detected by western blot assay. *P,0.05; **P,0.01.

**Fig 7:**
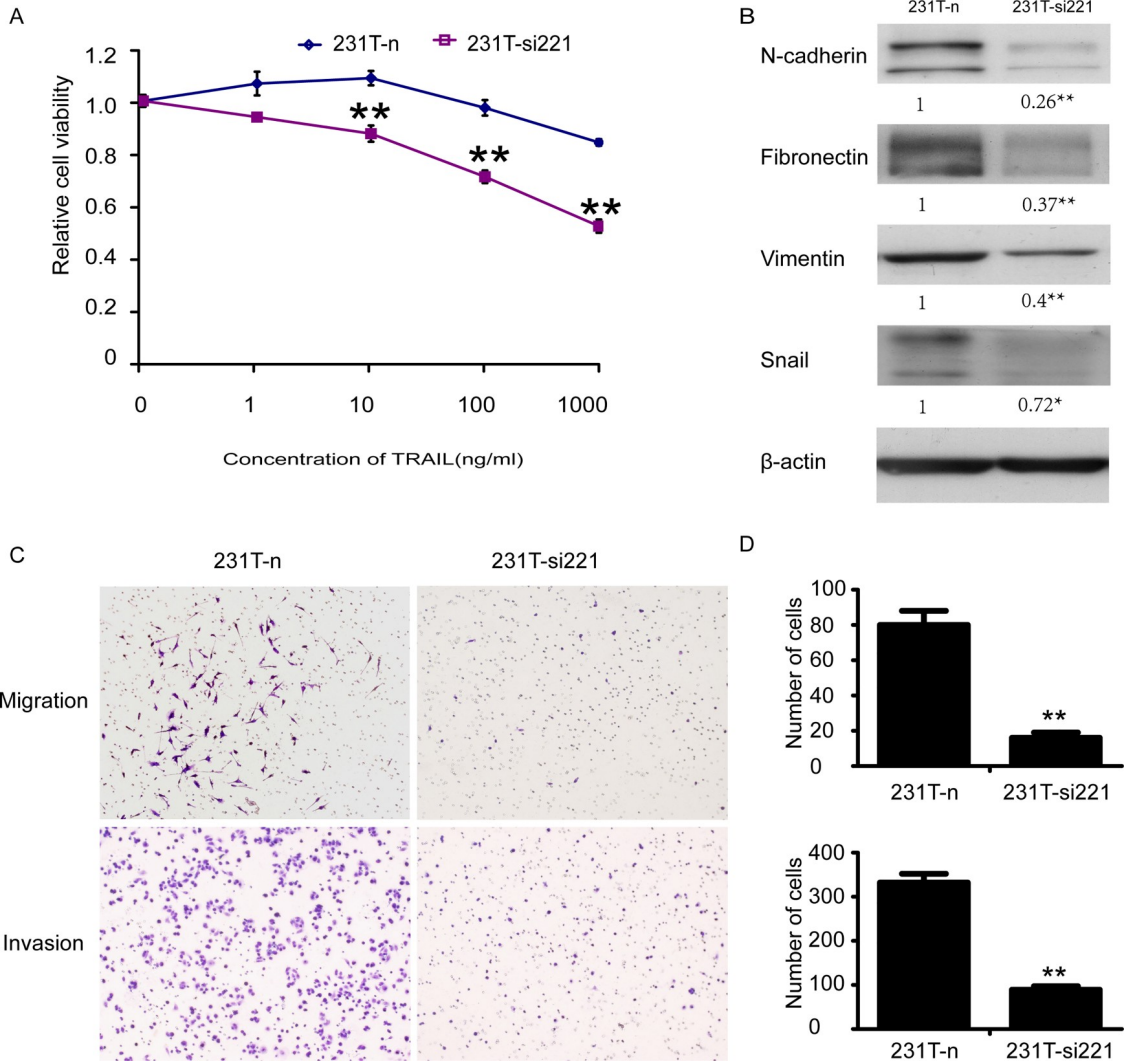
MiR-221 knockdown could sensitize 231T cells to TRAIL, reverse EMT and reduce cell mobility. **A.** Cultured 231T-n and 231Tsi221 cells with different concentrations of TRAIL in 96-well plates. 48 hours later examined their sensitivity to TRAIL by MTT assay. Points represented the average of three independent experiments. Bars stood for SD; **B.** The mesenchymal markers, N-cadherin, fibronectin, vimentin and Snail expressed in 231T-n and 231T-si221 cells were detected by western blot assay.b-actin was used as control; **C.** Migration and invasion assay of 231T-n cells and 231T-si221 cells. Staining the migrated cells with hematoxylin–eosin and count them in 10 representative fields under a light microscope. **D.** Summary graphs for migration and invasion were also shown, respectively. Data was presented as mean6SD. *P,0.05; **P,0.01.

## Supporting information

S1 File Raw data set. All underlying data for this study.
(ZIP)
