# Supplementary figures and images for: Correction: Trail Resistance Induces Epithelial-Mesenchymal Transition and Enhances Invasiveness by Suppressing PTEN via miR-221 in Breast Cancer
Source: PLoS One. 2019 Mar 21;14(3):e0214433. doi: 10.1371/journal.pone.0214433 (PMC6428298; doi:10.1371/journal.pone.0214433)

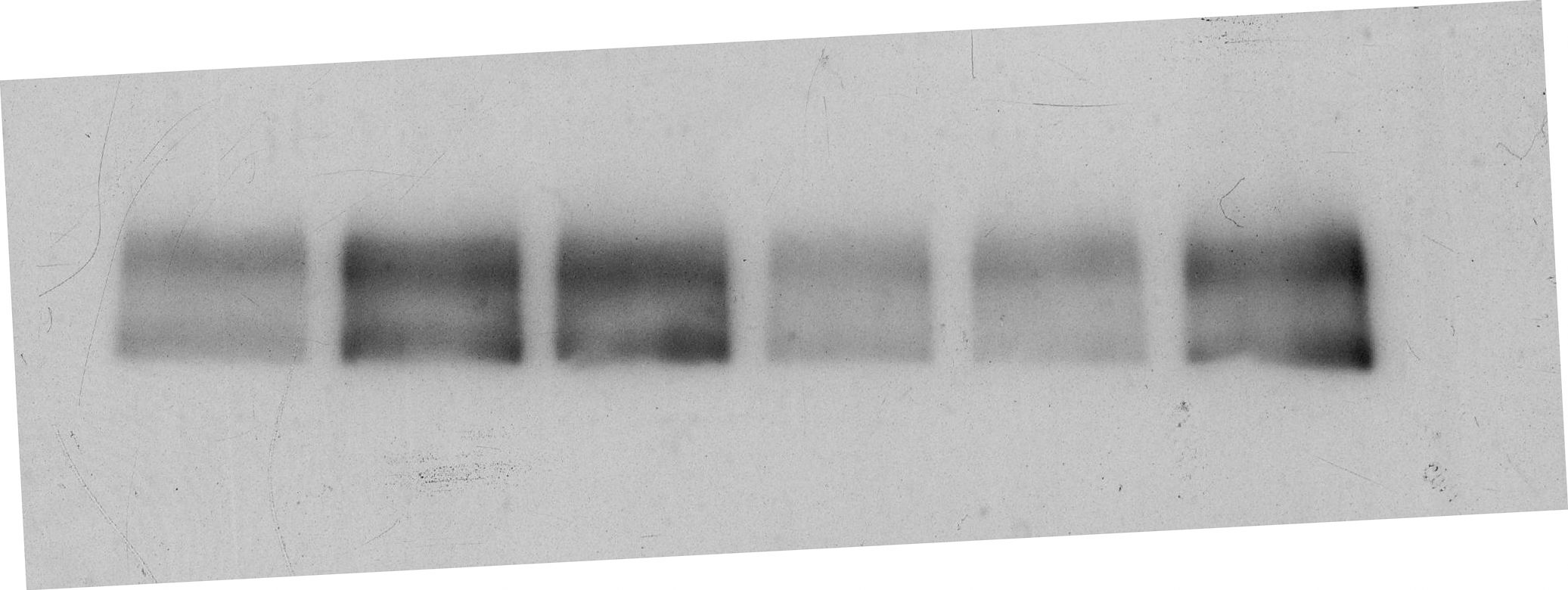

Supplement: S1 File — All underlying data for this study. (ZIP) [file pone.0214433.s001.zip › Raw Data/1. Fig 2A, 7B, 4D Fibronectin.jpg]

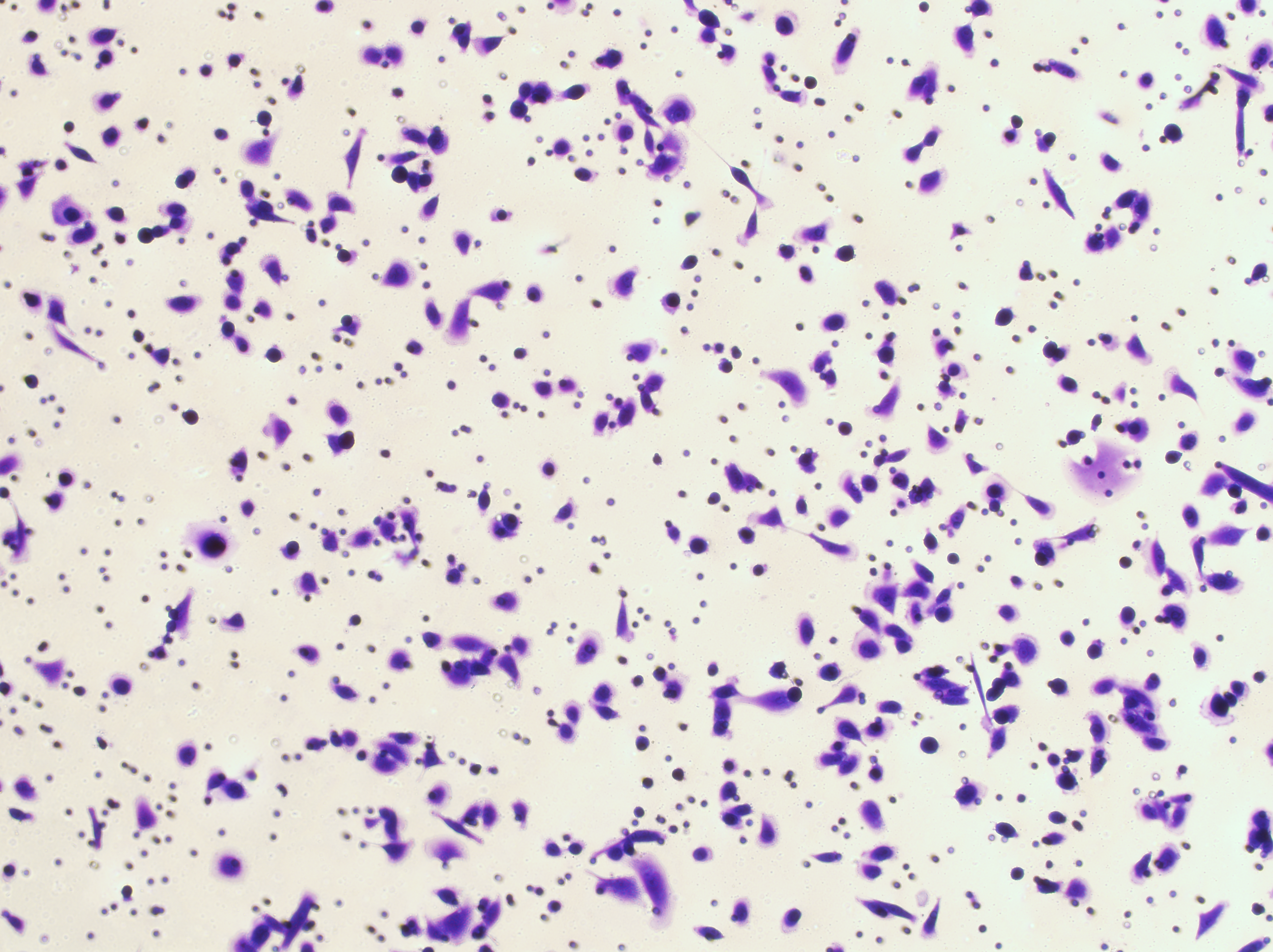

Supplement: S1 File — All underlying data for this study. (ZIP) [file pone.0214433.s001.zip › Raw Data/10. Fig 3C 231T.tif]

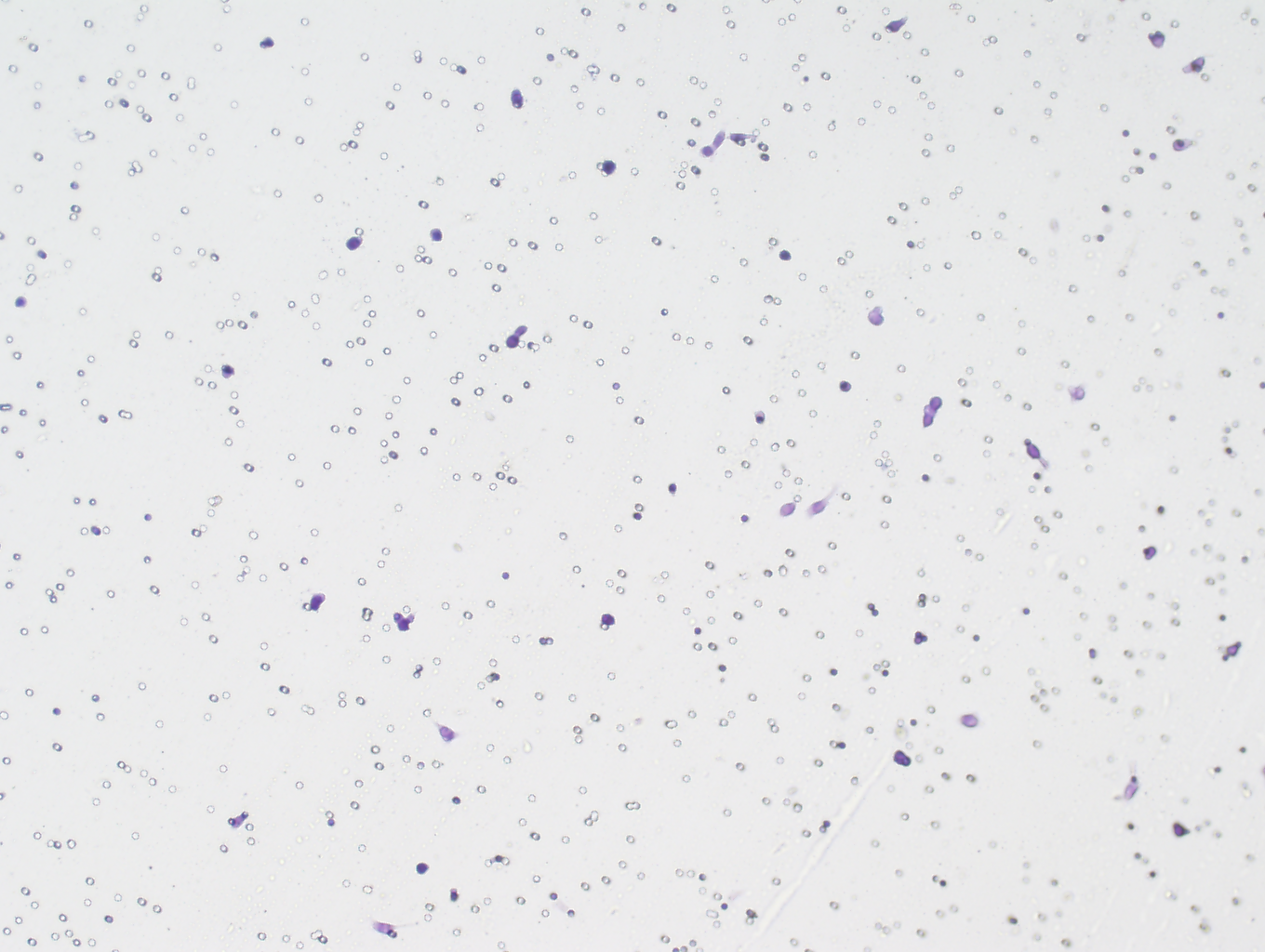

Supplement: S1 File — All underlying data for this study. (ZIP) [file pone.0214433.s001.zip › Raw Data/11. Fig 4C 231-n invasion.tif]

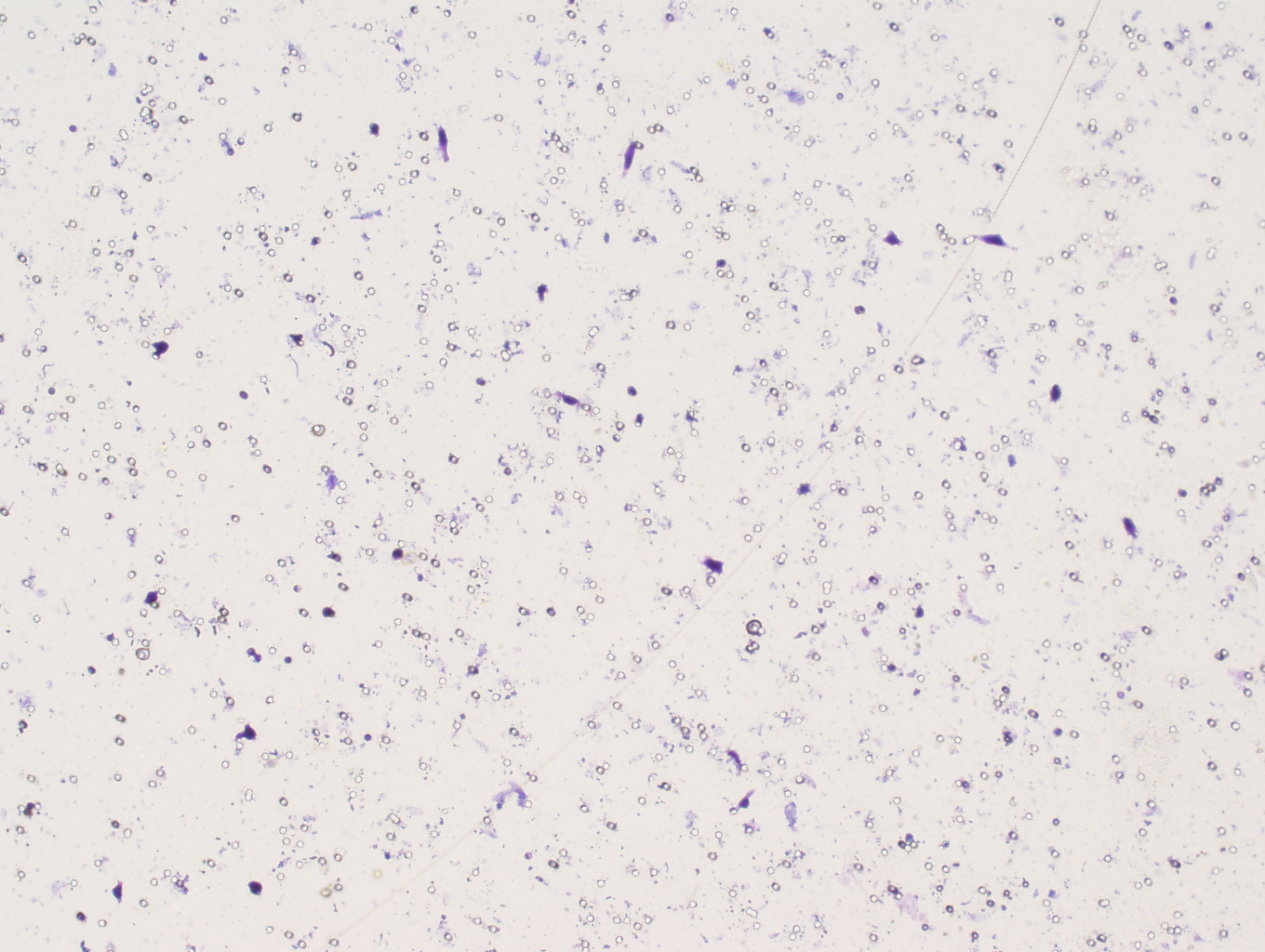

Supplement: S1 File — All underlying data for this study. (ZIP) [file pone.0214433.s001.zip › Raw Data/12. Fig 4C 231-n migration.tif]

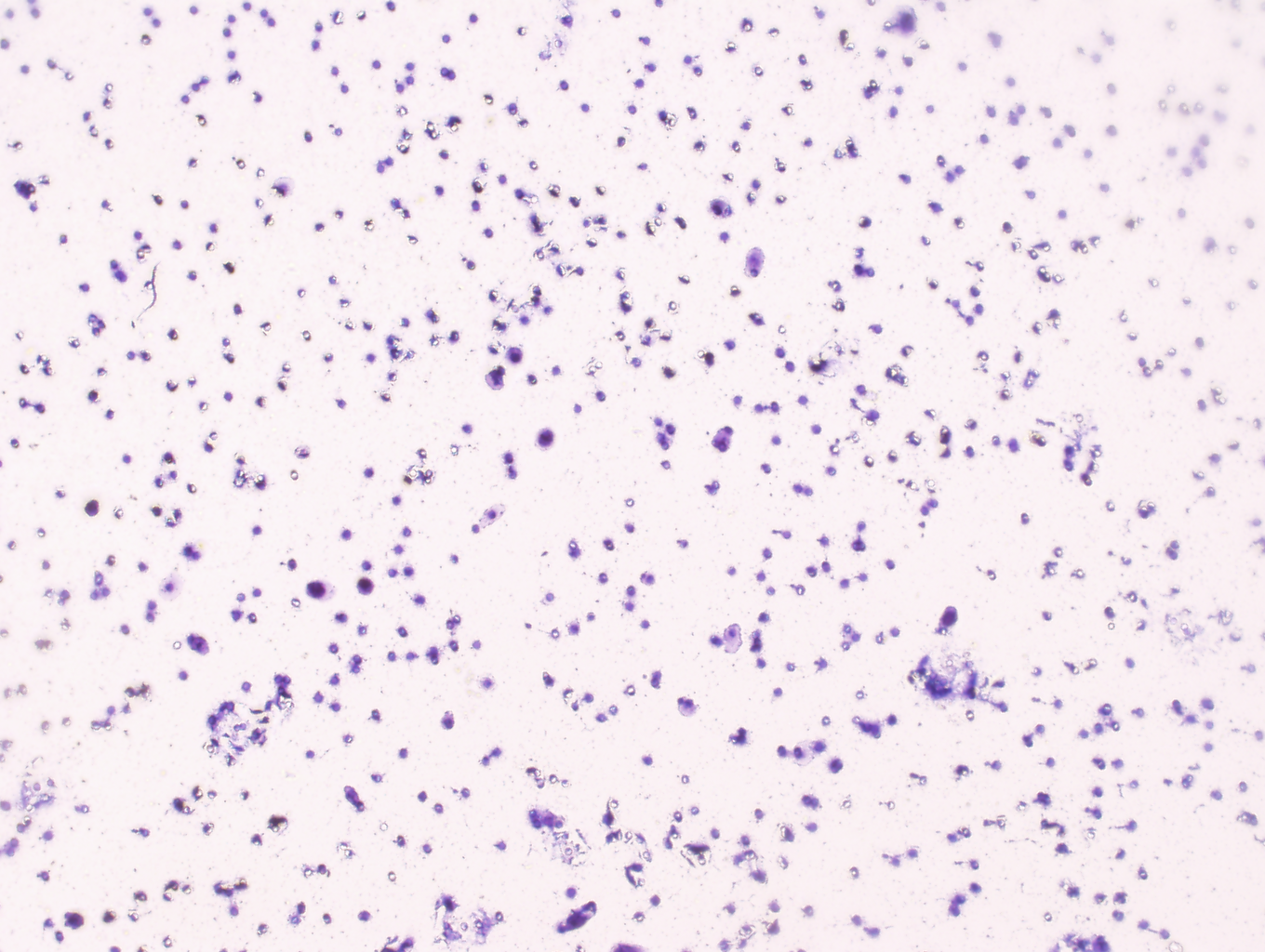

Supplement: S1 File — All underlying data for this study. (ZIP) [file pone.0214433.s001.zip › Raw Data/13. Fig 4C 231-siPTEN invasion.tif]

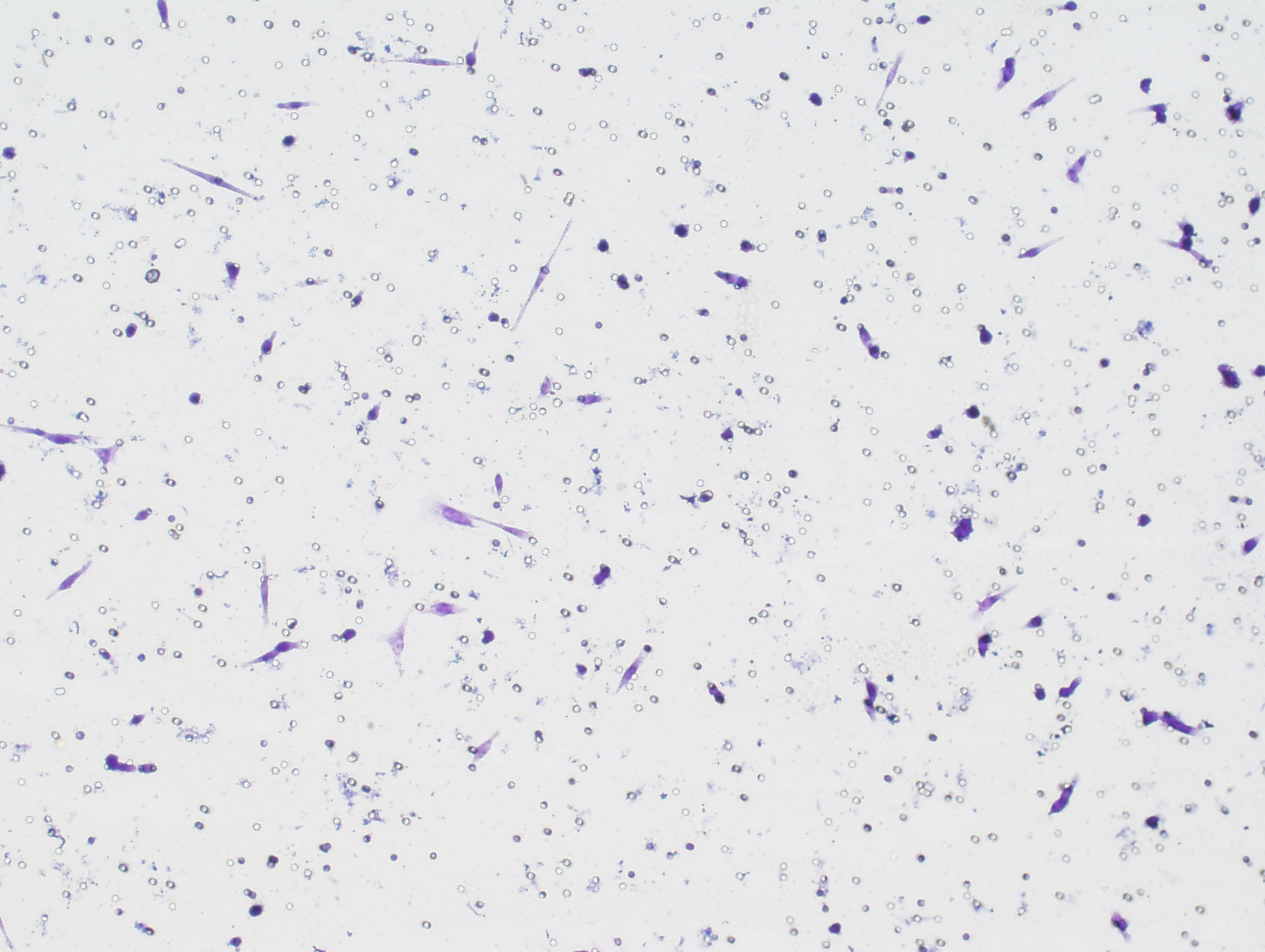

Supplement: S1 File — All underlying data for this study. (ZIP) [file pone.0214433.s001.zip › Raw Data/14. Fig 4C 231-siPTEN migration.tif]

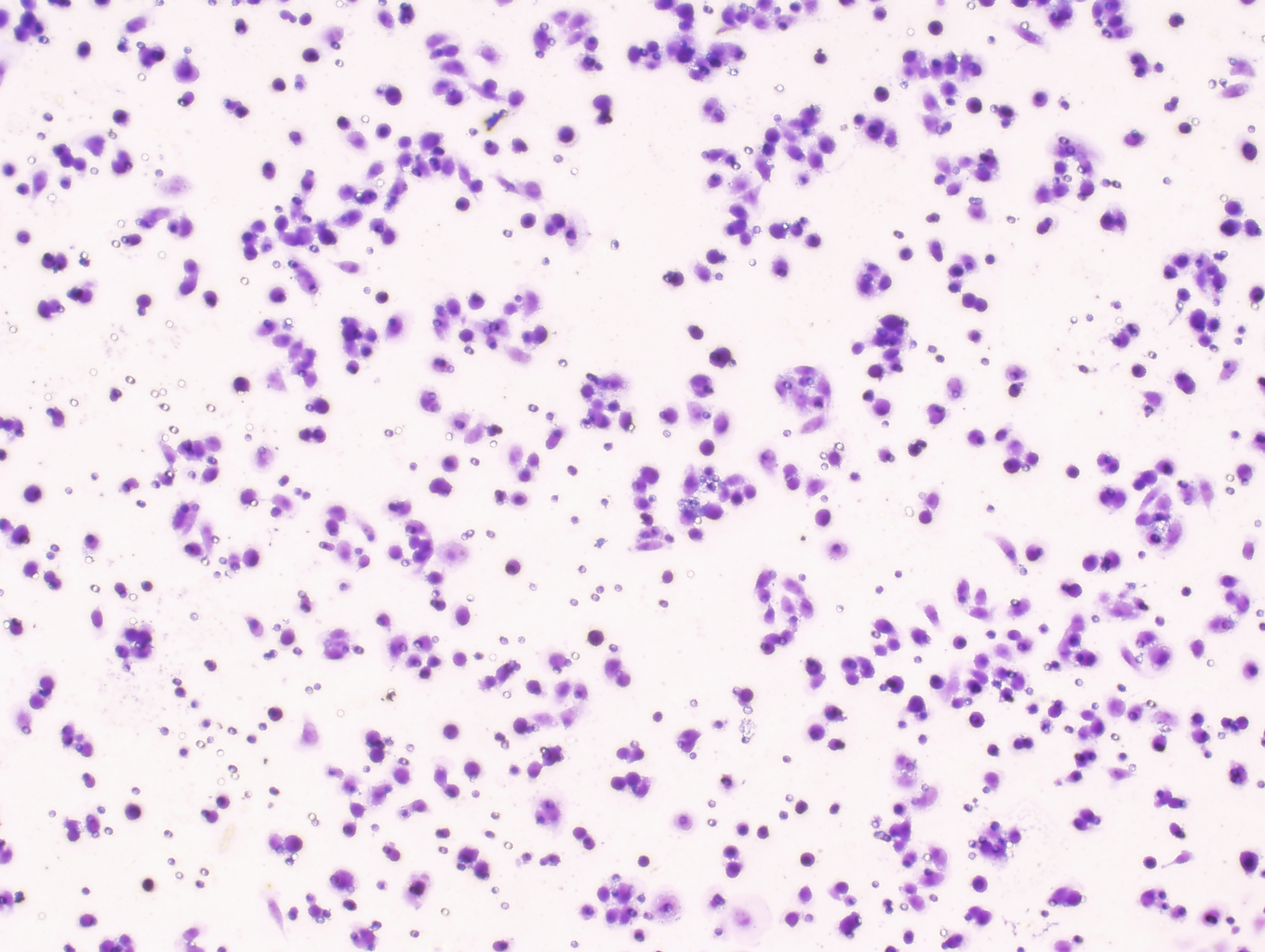

Supplement: S1 File — All underlying data for this study. (ZIP) [file pone.0214433.s001.zip › Raw Data/15. Fig 7C 231T-n invasion.tif]

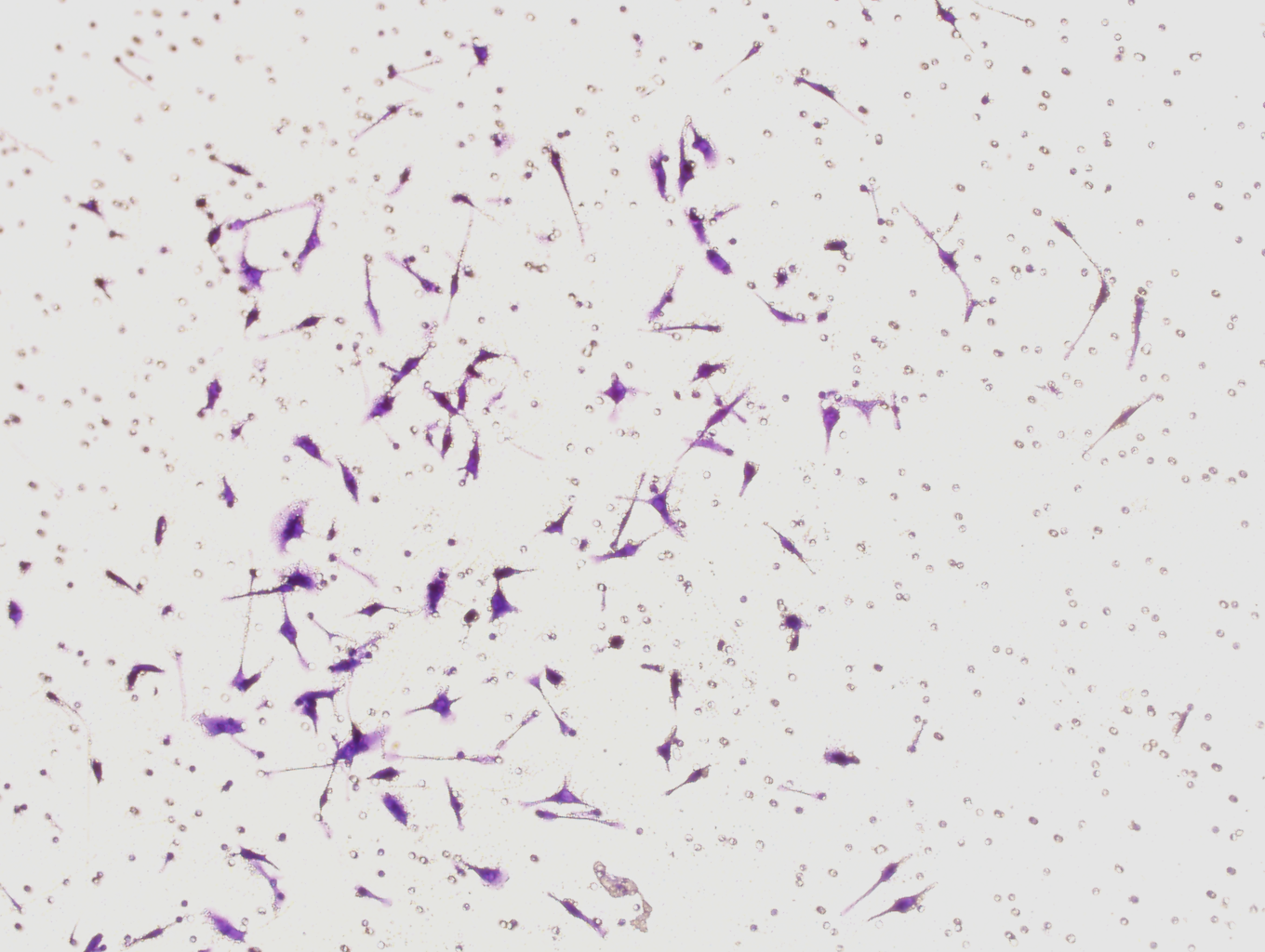

Supplement: S1 File — All underlying data for this study. (ZIP) [file pone.0214433.s001.zip › Raw Data/16. Fig 7C 231T-n migration.tif]

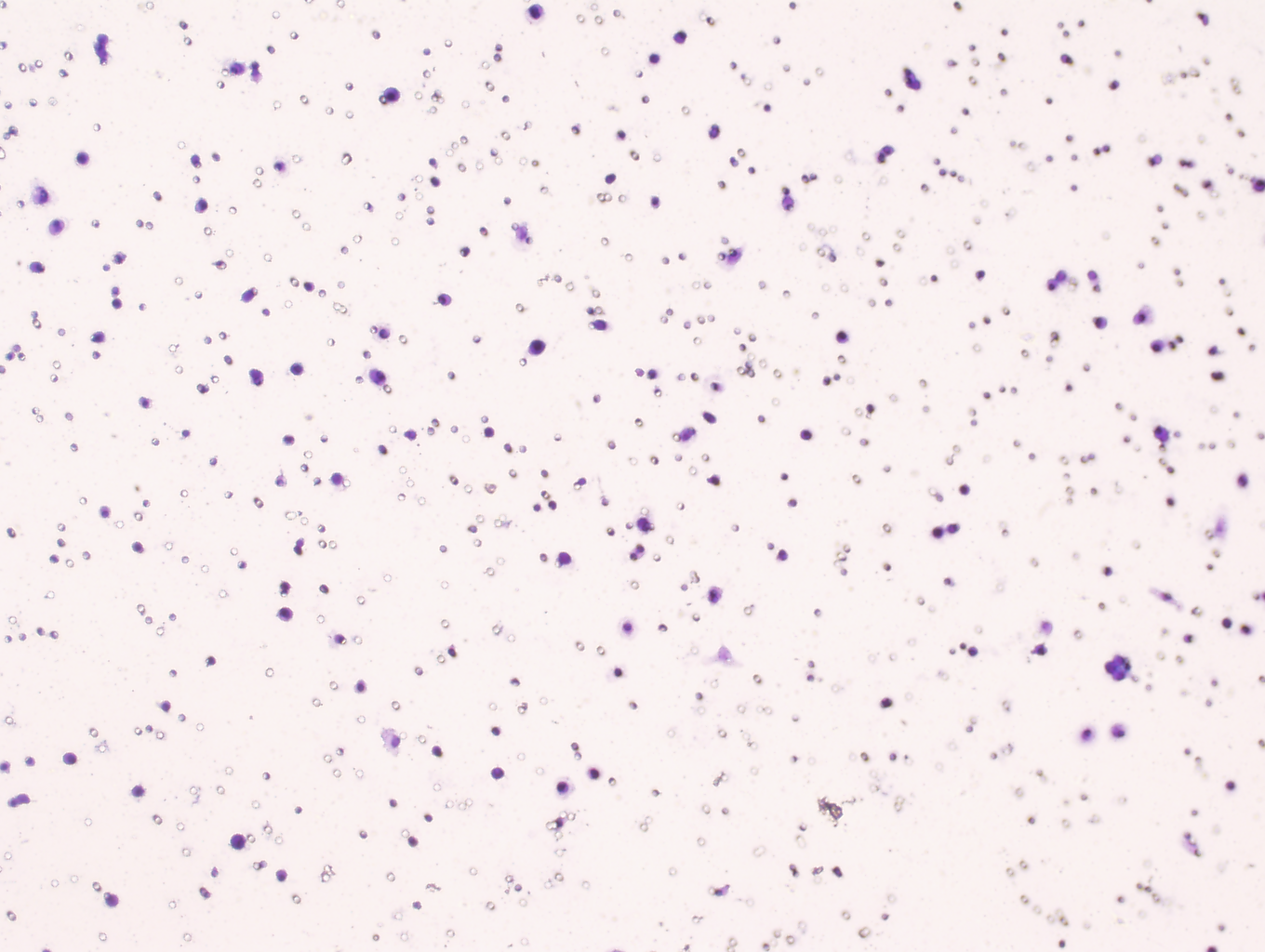

Supplement: S1 File — All underlying data for this study. (ZIP) [file pone.0214433.s001.zip › Raw Data/17. Fig 7C 231T-si221 invasion.tif]

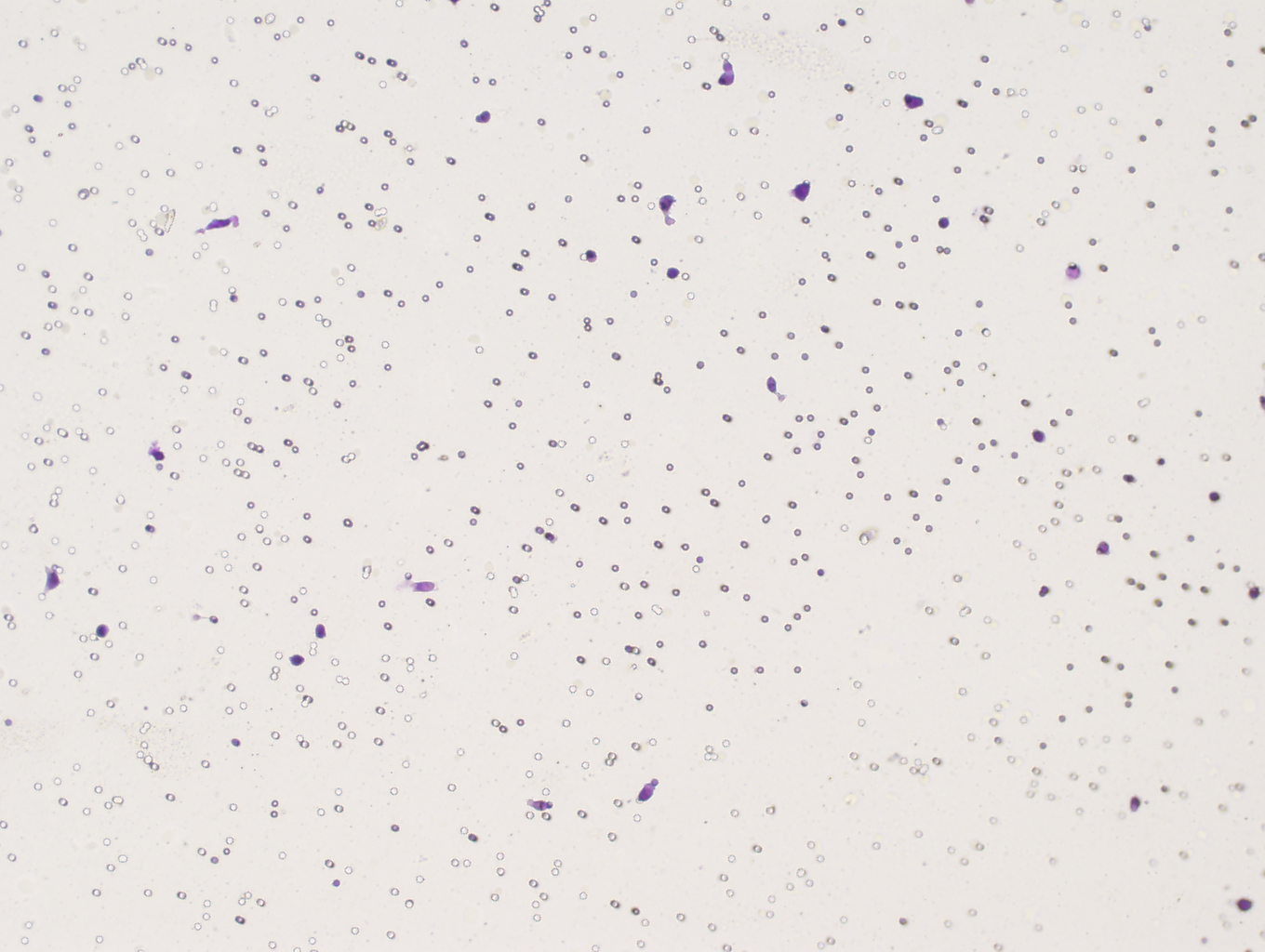

Supplement: S1 File — All underlying data for this study. (ZIP) [file pone.0214433.s001.zip › Raw Data/18. Fig 7C 231T-si221 migration.tif]

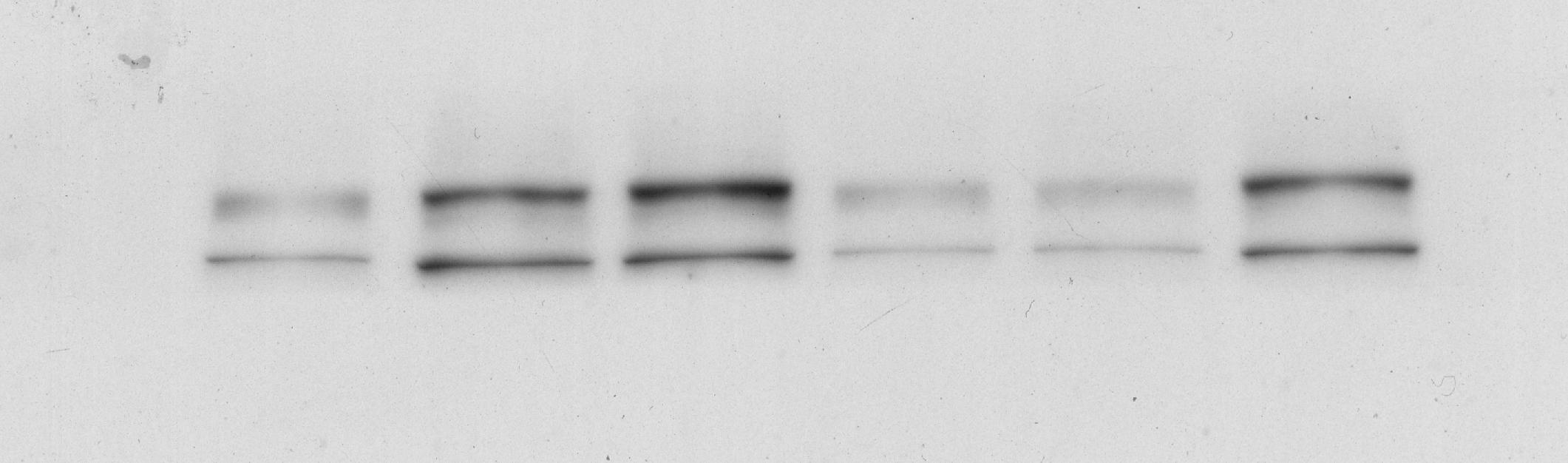

Supplement: S1 File — All underlying data for this study. (ZIP) [file pone.0214433.s001.zip › Raw Data/2. Fig 2A, 7B, 4D N-cadherin.jpg]

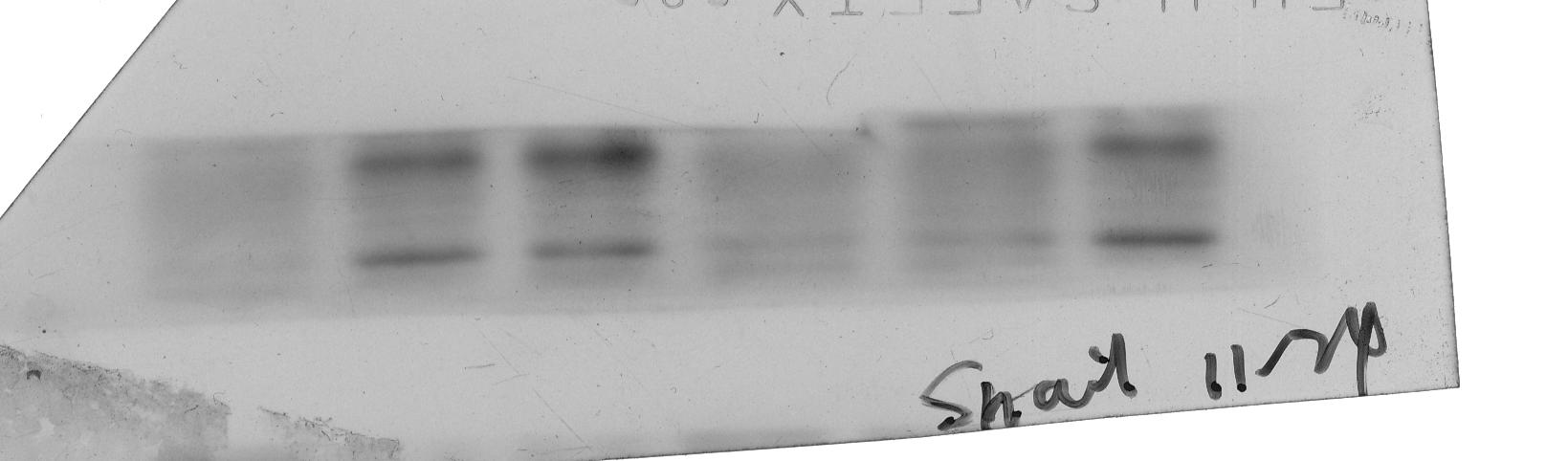

Supplement: S1 File — All underlying data for this study. (ZIP) [file pone.0214433.s001.zip › Raw Data/3. Fig 2A, 7B, 4D Snail.jpg]

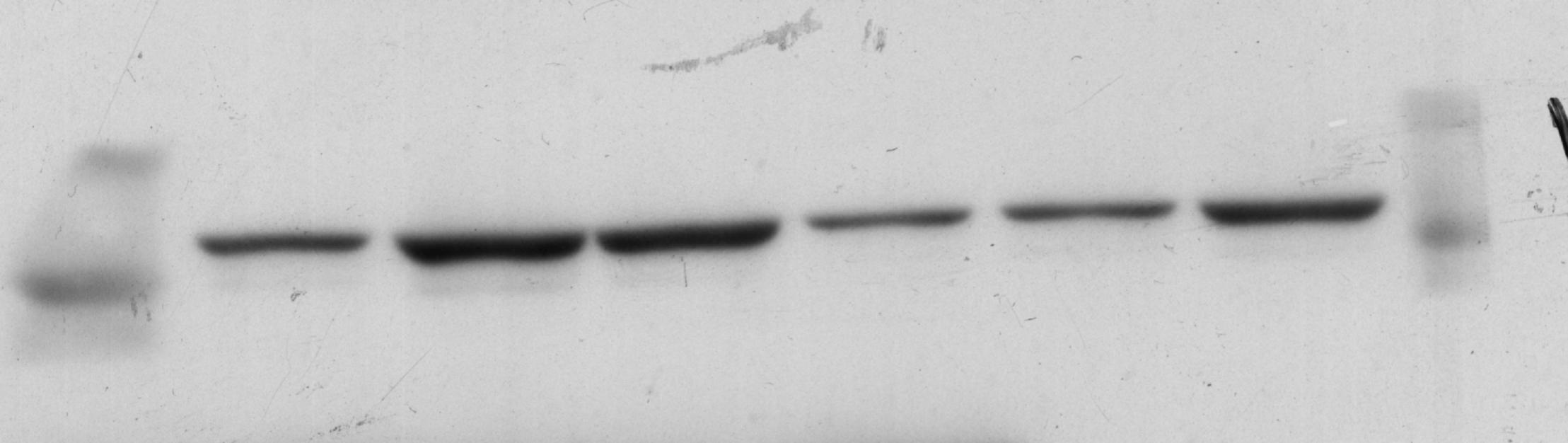

Supplement: S1 File — All underlying data for this study. (ZIP) [file pone.0214433.s001.zip › Raw Data/4. Fig 2A, 7B, 4D Vimentin.jpg]

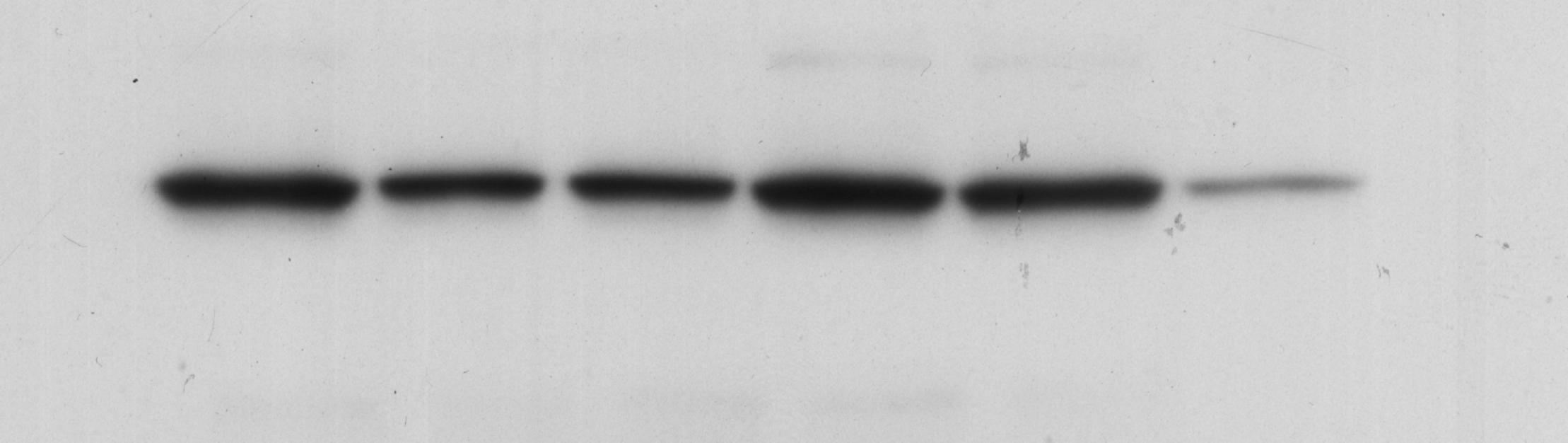

Supplement: S1 File — All underlying data for this study. (ZIP) [file pone.0214433.s001.zip › Raw Data/5. Fig 4A, 6C, 4D PTEN.jpg]

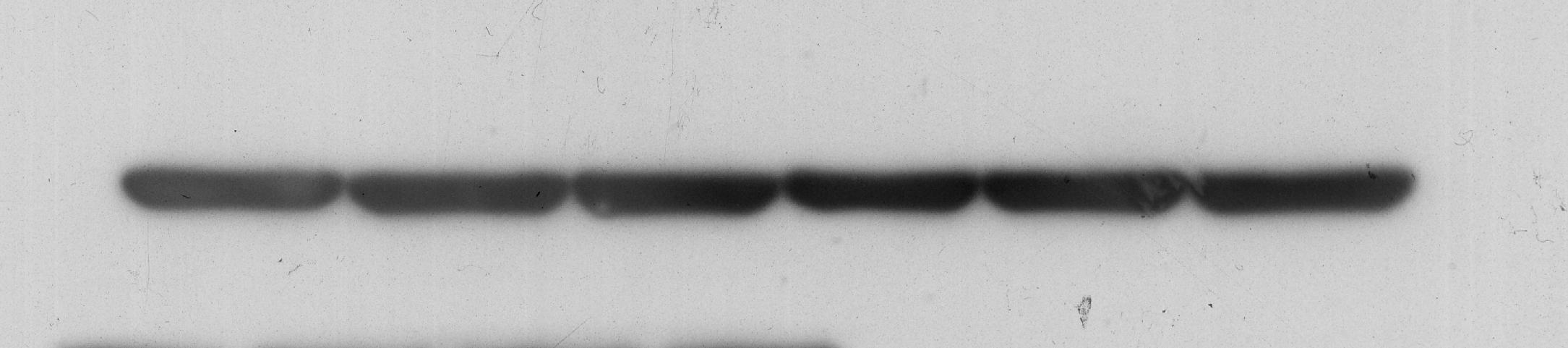

Supplement: S1 File — All underlying data for this study. (ZIP) [file pone.0214433.s001.zip › Raw Data/6. Fig 2A, 7B 4D a┬-actin.jpg]

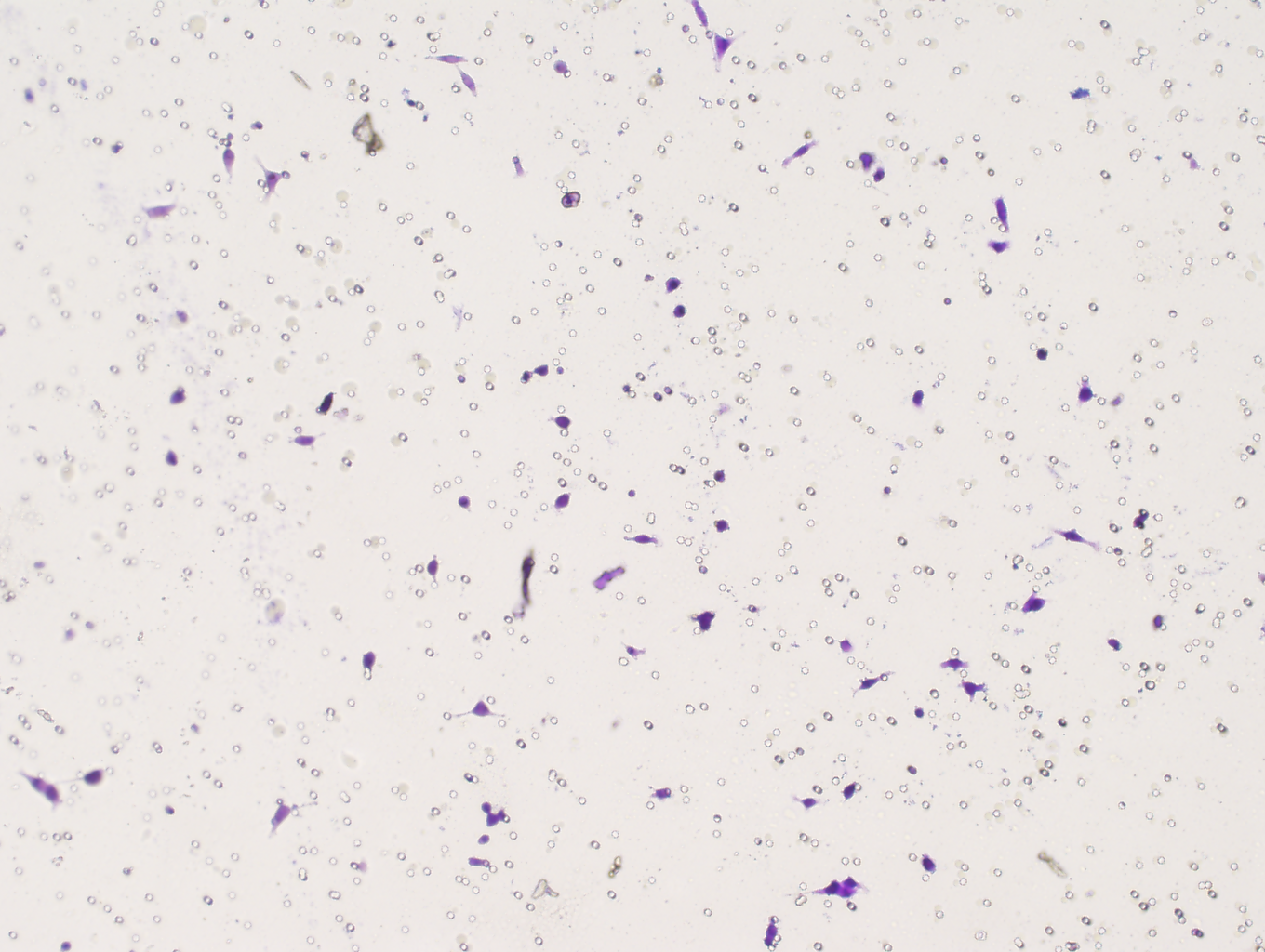

Supplement: S1 File — All underlying data for this study. (ZIP) [file pone.0214433.s001.zip › Raw Data/7. Fig 3A 231.tif]

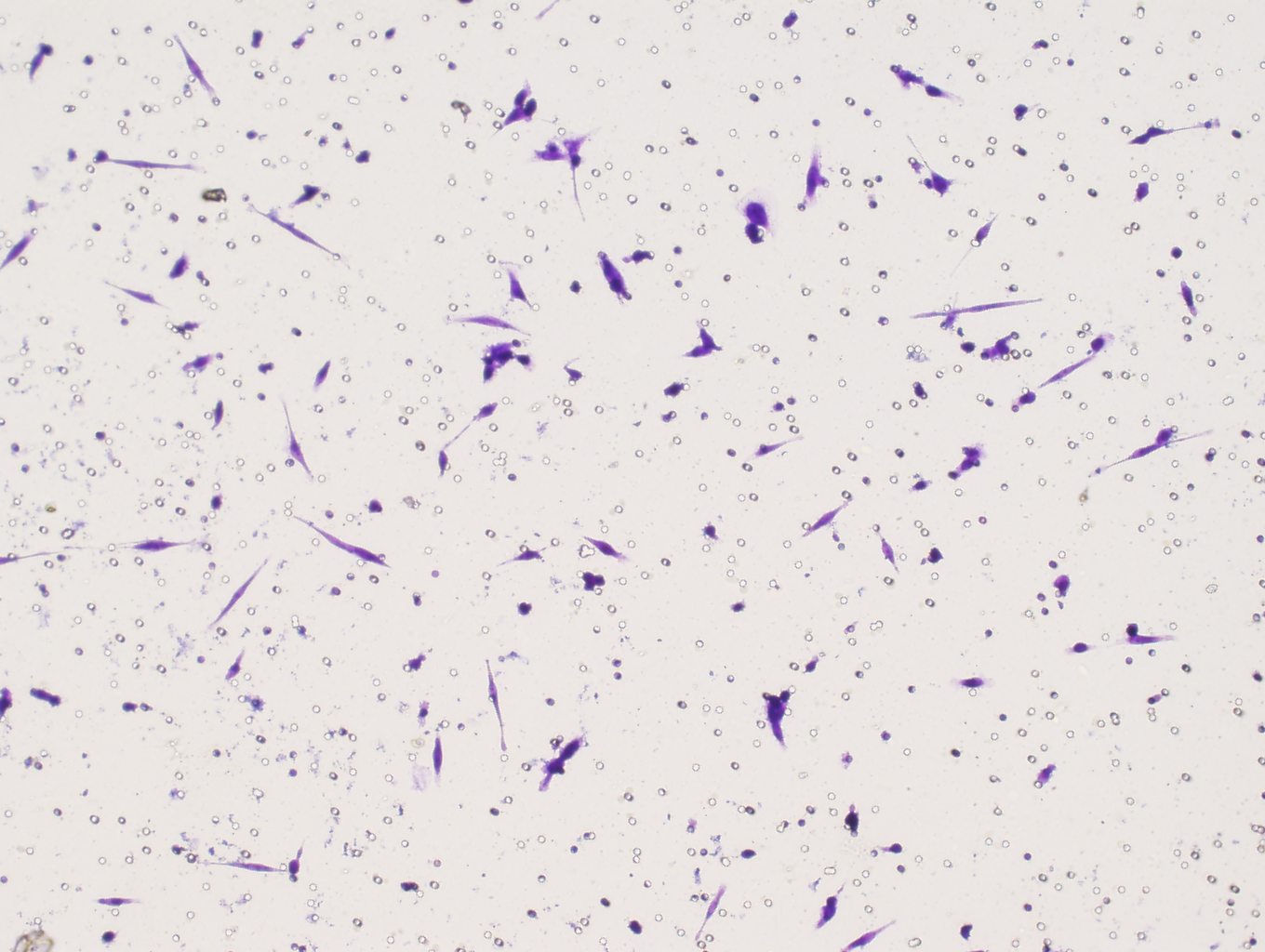

Supplement: S1 File — All underlying data for this study. (ZIP) [file pone.0214433.s001.zip › Raw Data/8. Fig 3A 231T.tif]

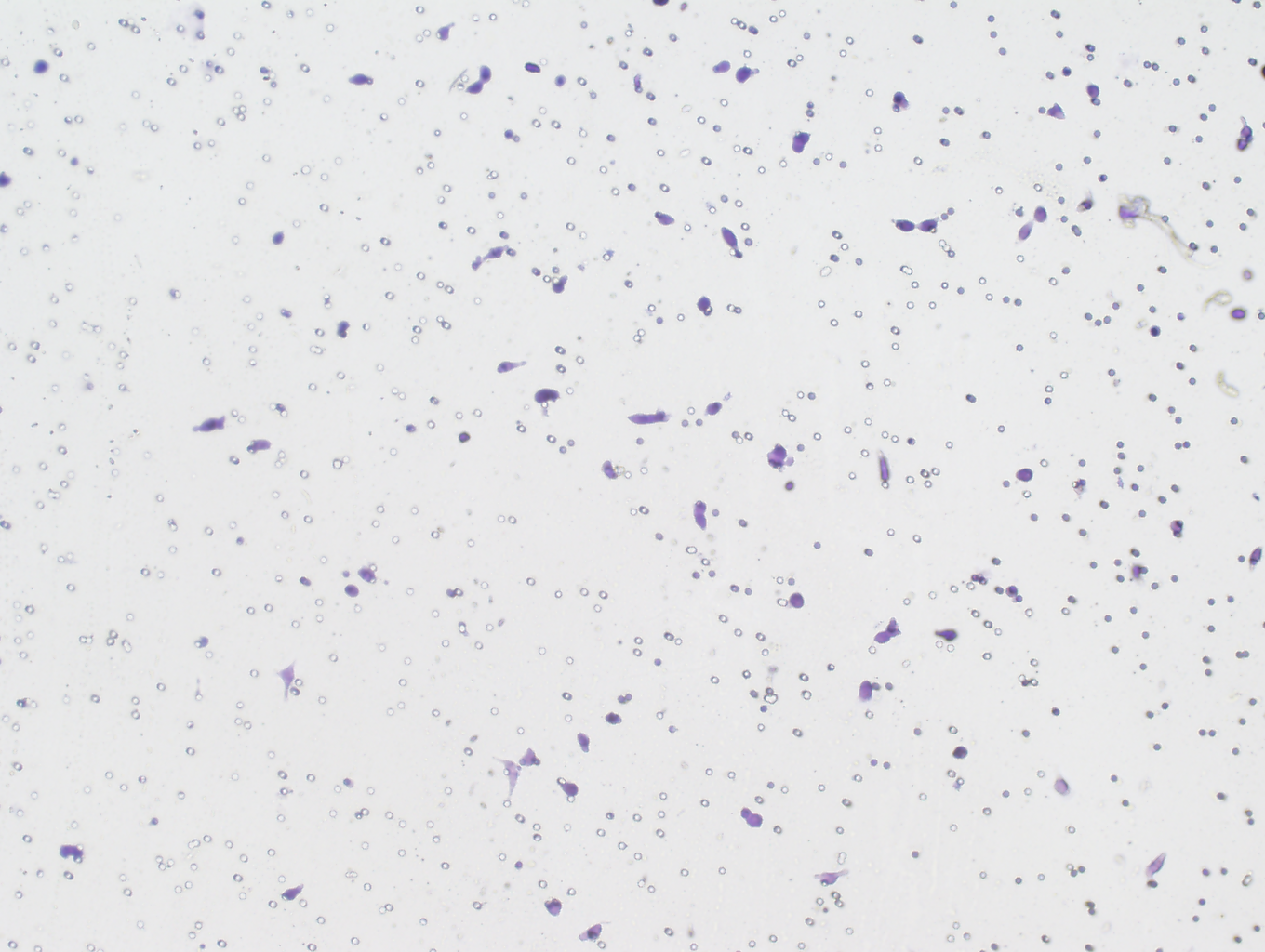

Supplement: S1 File — All underlying data for this study. (ZIP) [file pone.0214433.s001.zip › Raw Data/9. Fig 3C 231.tif]
